# Are environmental area characteristics at birth associated with overweight and obesity in school-aged children? Findings from the SLOPE (Studying Lifecourse Obesity PrEdictors) population-based cohort in the south of England

**DOI:** 10.1186/s12916-020-01513-0

**Published:** 2020-03-19

**Authors:** Sam Wilding, Nida Ziauddeen, Dianna Smith, Paul Roderick, Debbie Chase, Nisreen A. Alwan

**Affiliations:** 1 School of Primary Care, Population Sciences and Medical Education, Faculty of Medicine, University of Southampton, Southampton, UK; 2 School of Geography and Environmental Science, Faculty of Environmental and Life Sciences, University of Southampton, Southampton, UK; 3 Southampton City Council, Southampton, UK; 4 NIHR Southampton Biomedical Research Centre, University of Southampton and University Hospital Southampton NHS Foundation Trust, Tremona Road, Southampton, SO16 6YD UK

**Keywords:** Childhood overweight, Obesity, Area indices, Greenspace

## Abstract

**Background:**

Geographical inequalities in overweight and obesity prevalence among children are well established in cross-sectional research. We aimed to examine how environmental area characteristics at birth are related to these outcomes in childhood.

**Methods:**

Anonymised antenatal and birth data recorded by University Hospital Southampton linked to school-measured weight and height data for children within Southampton, UK, were utilised (14,084 children at ages 4–5 and 5637 at ages 10–11). Children’s home address at birth was analysed at the Lower and Middle layer Super Output Area (LSOA/MSOA) levels (areas with average populations of 1500 and 7000, respectively). Area-level indices (walkability, relative density of unhealthy food outlets, spaces for social interaction), natural greenspace coverage, supermarket density and measures of air pollution (PM_2.5_, PM_10_ and NO_*x*_) were constructed using ArcGIS Network Analyst. Overweight/obesity was defined as a body mass index (BMI; kg/m^2^) greater than the 85th centile for sex and age. Population-average generalised estimating equations estimated the risk of being overweight/obese for children at both time points. Confounders included maternal BMI and smoking in early pregnancy, education, ethnicity and parity. We also examined associations for a subgroup of children who moved residence between birth and outcome measurement.

**Results:**

There were mixed results between area characteristics at birth and overweight/obesity at later ages. MSOA relative density of unhealthy food outlets and PM_10_ were positively associated with overweight/obesity, but not among children who moved. LSOA greenspace coverage was negatively associated with the risk of being overweight/obese at ages 10–11 in all children (relative risk ratio 0.997, 95% confidence interval 0.995–0.999, *p* = 0.02) and among children who moved.

**Conclusions:**

Local access to natural greenspaces at the time of birth was inversely associated with becoming overweight or obese by age 10–11, regardless of migration. Increased access/protection of greenspace may have a role in the early prevention of childhood obesity.

## Background

Overweight and obesity among children are ongoing concerns for public health [1]. In 2016, 50 million girls and 74 million boys aged 5 to 19 years old were estimated to be affected by obesity worldwide, with the absolute and relative number increasing in all regions [2]. There is a growing yet inconsistent evidence base for rates of obesity being higher among children living in areas that experience high exposure to unhealthy food outlets [3], discourage physical activity through car-centric neighbourhood designs [4] and lack access to greenspace (natural land) [5]. This has led to bodies such as the World Health Organization to call for the creation of ‘health-promoting environments’ to address health inequalities [6].

Characteristics of areas that children are exposed to in utero and early life will shape their susceptibility to non-communicable diseases through epigenetic and behavioural adaptation [7, 8]. Proposed mechanisms include the setting of obesogenic dietary and physical activity habits influenced by the availability of calorie-dense foods and spaces for physical activity [3, 4]. Air pollution affects glucose tolerance in animal studies, which in turn affects the storage and expenditure of energy in later life, and similar processes likely occur in humans [9]. The diversity of the gut microbiota is affected by exposure to environmental pollutants and is associated with weight in later life [10]. Children with reduced access to greenspace report greater levels of anxiety and sadness, and this tracks into adolescence [11], which could lead to adaptations in appetite and energy regulation [12].

The majority of research linking early-life environmental area characteristics with weight in childhood is cross-sectional. As a result, it is unclear whether these associations are causal, an artefact of confounding or caused by selective migration; where families with fewer risk factors for their children becoming overweight or obese are more likely to move to areas which are advantaged [13]. Longitudinal research designs are better suited to address selective effects, as the risk factor can be measured before the outcome has developed. A recent systematic review found that there is a limited body of longitudinal research linking environmental area characteristics experienced in utero and in early life with subsequent childhood weight [14]. Factors associated with children becoming overweight/obese included air pollution (nitrogen oxides and particulate matter), neighbourhood disturbances (e.g. noisy neighbours, vandalism) and traffic exposure. Several factors which have been associated cross-sectionally with childhood overweight or obesity have yet to be tested longitudinally, for example, greenspace [5], walkability [4] and food outlets [3].

Studies included in the previous systematic review [14] did not state the mechanisms through which area characteristics affect weight in childhood and how these are related to confounders, which could result in confounding bias. Confounding occurs when estimating the effects of area characteristics [15], because socioeconomically disadvantaged populations are overrepresented within neighbourhoods with the greatest exposure to fast food outlets [16] and air pollution [17] and with the poorest access to greenspace [18].

We aimed to investigate the associations between environmental area characteristics (greenspace, walkability, supermarket density, unhealthy food outlet relative density, spaces for social interaction and air quality) measured around the home at birth with overweight/obesity at school age in a population-based cohort in the south of England, UK. To test the hypothesis that such exposures during pregnancy/early life are associated with overweight/obesity in childhood even if they significantly change nearer the time of outcome measurement, we restricted analysis to children who moved between birth and outcome measurement.

## Methods

### Individual-level data

SLOPE (Studying Lifecouse Obesity PrEdictors) is a linked population-based cohort of anonymised antenatal care and birth records to child health records for live singleton births (2003–2018) registered at the University Hospital Southampton (UHS) National Health Service (NHS) Foundation Trust, UK. It includes information collected at the first antenatal appointment (recommended to occur around 10 weeks into pregnancy [19]). Key measures self-reported at the appointment included maternal age, education, employment status, height, parity, ethnicity and smoking history. Maternal weight was measured by midwives and then converted into body mass index (BMI; kg/m^2^). Child’s weight and length of gestation were objectively measured at birth and converted into birthweight centiles using the latest available reference data for the UK [20]. The child’s home address was provided at Lower layer Super Output Areas (LSOA) level around the time of birth for families living in the Solent Community NHS Trust area (*n* = 38,147), which covers the area within and in the vicinity of the city of Southampton. This address is aggregated to the 2011 boundaries of LSOAs and Middle layer Super Output Areas (MSOAs) to maintain anonymity. LSOAs have an average population of 1500 and cover an average area of 4 km^2^ (standard deviation 15), whilst MSOAs have an average population of 7000 and cover an average area of 21 km^2^ (standard deviation 53). All environmental area characteristics were measured at the LSOA and MSOA levels to test if the associations differ over a larger area of exposure. One potential issue in any area-based analysis is the scale aspect of the modifiable areal unit problem (MAUP). This describes the situation where an association between two area or population-level characteristics may vary in statistical significance depending on the unit of analysis. Here, we consider this limitation by exploring the possible associations at two spatial scales [21].

### Outcome

We utilised child weight and height measured by school nurses at reception year (ages 4–5) and year 6 (ages 10–11), as part of the National Childhood Measurement Programme (NCMP) in England, with a parental opt-out option [22]. Participation rates in NCMP were 95% and 94% at ages 4–5 and 10–11, respectively, in England for the 2017/2018 school year and were slightly higher for Southampton [23]. Children’s BMI were converted to age- and sex-specific centiles, according to the 1990 UK weight reference [24]. Children are defined as overweight or obese if their BMI is greater than the 85th centile, in line with the guidance from the National Obesity Observatory and the population cut-off used in NCMP reports [23, 25].

### Environmental area characteristics

Several environmental area characteristics were collated for the purpose of this study, informed by previous systematic reviews on this topic [26]. These included greenspace (access to natural land), walkability, supermarket density, relative exposure to unhealthy food outlets, spaces for social interaction, particulate matter and nitrogen oxides.

Greenspace was measured using annual releases of the Ordnance Survey’s MasterMap Topography Layer (2006–2016) [27]. Greenspace areas were identified as natural environment polygons, with the exclusion of freshwater areas and marshes. The summary measure was defined as the proportion of the LSOA and MSOA covered by these polygons. The greenspace index was generated using each annual release, with births before 2006 assigned the value for 2006.

The walkability index was adapted from a US-based index which has been associated with BMI in adults [28] and is comprised of residential density, gradient change, intersection density and land-use mix. Residential density is the number of households in the area in the 2011 Census, per square kilometre [29]. Gradient change is the average slope (in degrees) between 5-m intervals, derived from Ordnance Survey’s OS Terrain 5 [30]. Intersection density is the number of road junctions with three or more exits per square kilometre, derived from the Ordnance Survey’s ITN Layer [31]. Land-use mix is an entropy score, where 0 indicates that the area is covered by one land-use type exclusively and 1 indicates that the area is perfectly split between all land-use types. The land-use types include residential, retail, office, entertainment, institutional and greenspace. Each component is expressed as a *z*-score relative to the county of Hampshire. The overall index is calculated as residential density − gradient change + intersection density × 2 + land-use mix. Intersection density is weighted twice, given the strong link between street connectivity and pedestrian activity in previous research [28]. Higher scores for the index indicate areas that are more walkable, and therefore encourage physical activity [28].

Supermarket density was derived from the Ordnance Survey’s Points of Interest dataset (2008–2017) [32]. Kernel density estimation was used, a technique that estimates the concentration of points within a given search radius, with a distance factor that gives greater weighting to points close to the centre [33]. Kernel density estimates were generated for a radius of 800 m around each supermarket in each year, at 100-m intervals. These estimates were then averaged across each LSOA/MSOA. We chose to keep supermarkets separate from unhealthy and healthy food indices, because supermarkets sell food that enables both healthy and unhealthy diets. This stance is supported by the inconsistent link between supermarket access and obesity in research [34]. Births before 2008 were assigned the value for 2008.

Exposure to unhealthy food outlets was also derived from the Ordnance Survey’s Points of Interest dataset (2008–2017) [32]. As above, kernel density estimates were generated separately each year for unhealthy (bakeries, confectioners, convenience stores and takeaways) and healthy (grocers, markets and health food stores) food outlets. The densities for both types of outlets are averaged across the area; any of the relative exposure to unhealthy outlets is calculated by subtracting the density of healthy outlets from that of unhealthy outlets, in line with a previous study [35]. Positive values indicate a greater exposure to unhealthy outlets and vice versa.

Spaces for social interaction were places that we felt would encourage young families to socially network, which may involve activities or walking to those places for the child. Our selection was informed by a range of sources including recommendations on the Mumsnet internet forums (https://www.mumsnet.com/Talk) for places that offer classes for young children or public spaces where young families could meet. In addition, from the Points of Interest data, cafes, community centres, gymnasiums/leisure centres, libraries, places of worship, playgrounds, soft play centres and swimming pools were selected. Data on opening and closing dates for children’s centres (e.g. ‘Sure Start centres’) were derived from the UK Government’s ‘get information about schools’ service [36] in April 2019. Similar to the food indices, kernel density estimates were created for each year for the total sum of spaces for social interaction and averaged across the area. Births before 2008 were assigned the value for 2008.

Three air quality components (particulate matter (PM)_2.5_, PM_10_ and nitrogen oxides (NO_*x*_)) were used, based on previous evidence of their association with being overweight or obese in childhood [14]. These indicators are provided as annual means for 2003–2017 at a 1 km × 1 km resolution [37] and were converted into weighted averages for each LSOA and MSOA in respective years. Further detail is available on www.southampton.ac.uk/slope/data/area-data.page.

### Sample

**Fig. 1:**
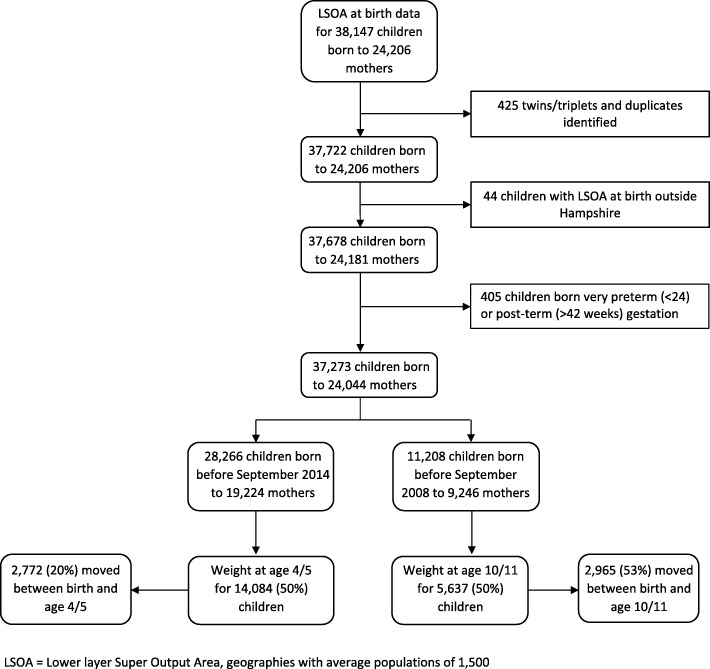
Sample selection diagram

Derivation of the sample for this analysis is outlined in Fig. 1. Thirty-eight thousand one hundred forty-seven UHS records included in SLOPE were for children within the Solent Community NHS Trust area, which provided LSOA at birth. Four hundred twenty-five twins, triplets and duplicate records were excluded; a further 44 records were excluded for having an LSOA outside of Hampshire. A further 405 records were excluded for being born before 24 weeks gestation or after 42 weeks gestation. Children born before September 2014 were old enough to be measured at schools at age 4/5, which amounted to a linked sample of 28,226 children (73.9%). Of these children, 14,084 had a measurement for weight in the linked dataset at age 4/5 (50.0%), and 2772 (19.7%) of these changed home LSOA between birth and measurement. Children born before September 2008 were old enough to be measured at schools at age 10/11, which amounted to a sample of 11,208 children (30.1%). Of these children, 5637 had a measurement for weight at age 10/11 (50.3%), of which 2965 (53.0%) changed home LSOA between birth and measurement.

### Statistical analysis

**Fig. 2:**
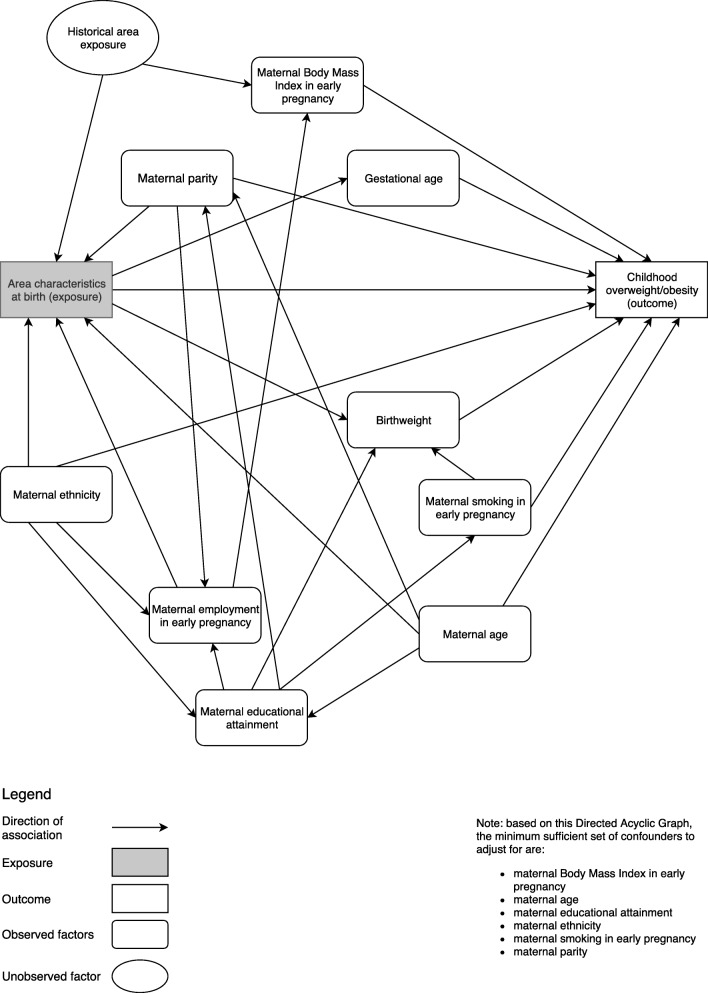
Directed acyclic graph illustrating the relationship between area characteristics and weight in childhood

We constructed a directed acyclic graph (DAG) using DAGitty v2.5 [38] which illustrates the assumptions made in this analysis of area effects on weight in childhood, based on previous research (Fig. 2). We assumed that area characteristics such as air quality affect gestational age [39] and birthweight [40] and independently affect weight in childhood [41] through epigenetic and behavioural adaptation. Area exposure at birth is affected by maternal ethnicity, employment, parity, age and education, because disadvantaged populations tend to reside in the most challenging environments [16–18]. The types of areas mothers lived in during pregnancy will have affected their BMI [3], and their children will likely be born into areas with similar characteristics. Based on this DAG, the minimum sufficient set of confounders [42] to include are maternal BMI, age, education, ethnicity, smoking at the start of pregnancy and parity. Models for each area characteristic were tested separately, adjusted for this core set of confounders.

Population-average generalised estimating equations are used to adjust for clustering by LSOA and MSOA in separate models [43]. These models estimate the relative risk for each area characteristic/index and the risk of children being overweight or obese as a binary variable at ages 4–5 and 10–11. All area characteristics are presented with unadjusted and adjusted (for maternal individual-level characteristics) relative risk and are tested at the LSOA and MSOA levels. In sensitivity analyses, we then assess if the associations were similar for children who lived in different areas (LSOAs) at measurement and birth, as significant associations in this sub-sample would demonstrate that early-life area characteristics may have long-term effects, whilst for children who have not moved, the association could be due to exposure at the same time of developing overweight/obesity. In a sensitivity analysis for the year 6 outcome, we conducted multiple imputation to address any potential bias caused by missingness of the outcome variable. Statistical analyses were conducted in Stata version 15.1 [44].

## Results

Table 1 displays the characteristics of the sample with outcome data. At age 4–5, 23.1% of children were overweight or obese, and this increased to 34.6% at age 10–11. Among the maternal characteristics, never smoking, university education, primiparity and being employed at the start of pregnancy were more common among children who were not affected by overweight/obesity. Maternal obesity in early pregnancy was almost twice as prevalent for children aged 4–5 who were affected by overweight or obesity (29.5%), in comparison with children who were not (16.6%), and a similar trend is present in children aged 10–11. Birthweight centile was higher among children who were overweight or obese.

**Table 1:** Sample characteristics at birth by childhood overweight/obesity status

	Age 4–5 years (*n* = 14,084)		Age 10–11 years (*n* = 5637)		Missing (%)
	Not overweight or obese, < 85th centile	Overweight/obese, ≥ 85th centile	Not overweight or obese, < 85th centile	Overweight/obese, ≥ 85th centile	
Total	10,826 (76.9%)	3258 (23.1%)	3687 (65.4%)	1950 (34.6%)	0 (0.0%)
Maternal BMI at booking (kg/m^2^)					63 (0.4%)
Underweight (< 18.5)	429 (4.0%)	56 (1.7%)	161 (4.4%)	36 (1.9%)	
Normal weight (18.5–24.9)	5978 (55.2%)	1286 (39.5%)	2251 (61.1%)	844 (43.3%)	
Overweight (25.0–29.9)	2627 (24.3%)	956 (29.3%)	828 (22.5%)	554 (28.4%)	
Obese (> 30.0)	1792 (16.6%)	960 (29.5%)	447 (12.1%)	516 (26.5%)	
Maternal age mean (SD)	27.6 (5.9)	27.3 (6.0)	27.1 (6.0)	27.0 (6.1)	0 (0.0%)
Maternal smoking at booking					63 (0.4%)
Never smoker	5324 (49.4%)	1393 (42.9%)	1785 (48.6%)	838 (43.2%)	
Ex-smoker	3288 (30.5%)	1004 (30.9%)	1064 (29.0%)	560 (28.9%)	
Up to 10 cigarettes per day	1291 (12.0%)	503 (15.5%)	444 (12.1%)	287 (14.8%)	
10–20 cigarettes per day	816 (7.6%)	314 (9.7%)	353 (9.6%)	237 (12.2%)	
> 20 cigarettes per day	68 (0.6%)	33 (1.0%)	25 (0.7%)	18 (0.9%)	
Maternal ethnicity					0 (0.0%)
White	8735 (80.7%)	2645 (81.2%)	2939 (79.7%)	1483 (76.1%)	
Mixed	140 (1.3%)	58 (1.8%)	40 (1.1%)	39 (2.0%)	
Asian	1021 (9.4%)	284 (8.7%)	284 (7.7%)	176 (9.0%)	
Black/African/Caribbean	222 (2.1%)	95 (2.9%)	45 (1.2%)	47 (2.4%)	
Others	214 (2.0%)	53 (1.6%)	70 (1.9%)	37 (1.9%)	
Not known	494 (4.6%)	123 (3.8%)	309 (8.4%)	168 (8.6%)	
Maternal highest education at booking					25 (0.2%)
University	2301 (21.3%)	518 (15.9%)	647 (17.6%)	232 (11.9%)	
College	4350 (40.2%)	1348 (41.4%)	1366 (37.1%)	752 (38.6%)	
Up to secondary school	4162 (38.5%)	1388 (42.7%)	1667 (45.3%)	962 (49.4%)	
Maternal employment at booking					165 (1.0%)
Unemployed	4110 (38.4%)	1343 (41.6%)	1446 (39.6%)	832 (43.1%)	
Maternal parity at booking					0 (0.0%)
No previous live births	4815 (44.5%)	1329 (40.8%)	1662 (45.1%)	815 (41.8%)	
One previous live birth	3693 (34.1%)	1112 (34.1%)	1230 (33.4%)	645 (33.1%)	
Two previous live births	1459 (13.5%)	496 (15.2%)	512 (13.9%)	295 (15.1%)	
Three or more previous live births	859 (7.9%)	321 (9.9%)	283 (7.7%)	195 (10.0%)	
Birthweight centile mean (SD)	47 (29.1)	56 (29.3)	47 (29.4)	52 (29.8)	
Did not change home LSOA	8717 (77.1%)	2109 (76.1%)	1771 (48.0%)	901 (46.2%)	0 (0.0%)
Changed home LSOA	2595 (22.9%)	663 (23.9%)	1916 (52.0%)	1049 (53.8%)	
Area measures					
Greenspace % LSOA (SD)	26.0 (19.0)	26.2 (19.1)	27.8 (19.9)	26.3 (18.7)	0 (0.0%)
Greenspace % MSOA (SD)	29.0 (17.7)	28.7 (17.3)	30.8 (18.6)	28.7 (17.5)	0 (0.0%)
Walkability LSOA (SD)	1.7 (2.8)	1.8 (2.7)	1.5 (2.8)	1.7 (2.7)	0 (0.0%)
Walkability MSOA (SD)	2.8 (2.7)	2.9 (2.7)	2.7 (2.8)	2.9 (2.6)	0 (0.0%)
Supermarket density LSOA (SD)	1.0 (0.9)	1.0 (1.0)	1.0 (0.9)	0.9 (0.9)	0 (0.0%)
Supermarket density MSOA (SD)	0.9 (0.7)	0.9 (0.7)	0.9 (0.7)	0.9 (0.7)	0 (0.0%)
Unhealthy food index LSOA (SD)	6.2 (6.4)	6.3 (6.5)	4.2 (3.6)	4.4 (3.6)	0 (0.0%)
Unhealthy food index MSOA (SD)	5.4 (4.4)	5.4 (4.4)	3.8 (2.4)	4.0 (2.4)	0 (0.0%)
Spaces for social interaction LSOA (SD)	7.3 (5.2)	7.5 (5.1)	6.8 (4.8)	7.1 (4.9)	0 (0.0%)
Spaces for social interaction MSOA (SD)	6.6 (3.9)	6.8 (3.9)	6.2 (3.8)	6.6 (3.8)	0 (0.0%)
PM_2.5_ LSOA (SD)	13.2 (1.5)	13.1 (1.5)	12.8 (1.2)	12.8 (1.2)	0 (0.0%)
PM_2.5_ MSOA (SD)	13.1 (1.5)	13.1 (1.4)	12.8 (1.2)	12.8 (1.2)	0 (0.0%)
PM 10 LSOA (SD)	18.6 (2.4)	18.5 (2.3)	19.8 (1.5)	19.9 (1.5)	0 (0.0%)
PM_10_ MSOA (SD)	18.6 (2.3)	18.5 (2.3)	19.7 (1.5)	19.8 (1.5)	0 (0.0%)
NO_*x*_ LSOA (SD)	40.2 (12.7)	40.2 (12.9)	31.8 (6.0)	32.1 (6.2)	0 (0.0%)
NO_*x*_ MSOA (SD)	40.2 (12.5)	40.0 (12.4)	31.4 (5.5)	31.6 (5.5)	0 (0.0%)

*LSOA* areas with average populations of 1500 and an area of 4 km^2^, *MSOA* areas with average populations of 7000 and an area of 21 km^2^, *SD* standard deviation

**Fig. 3:**
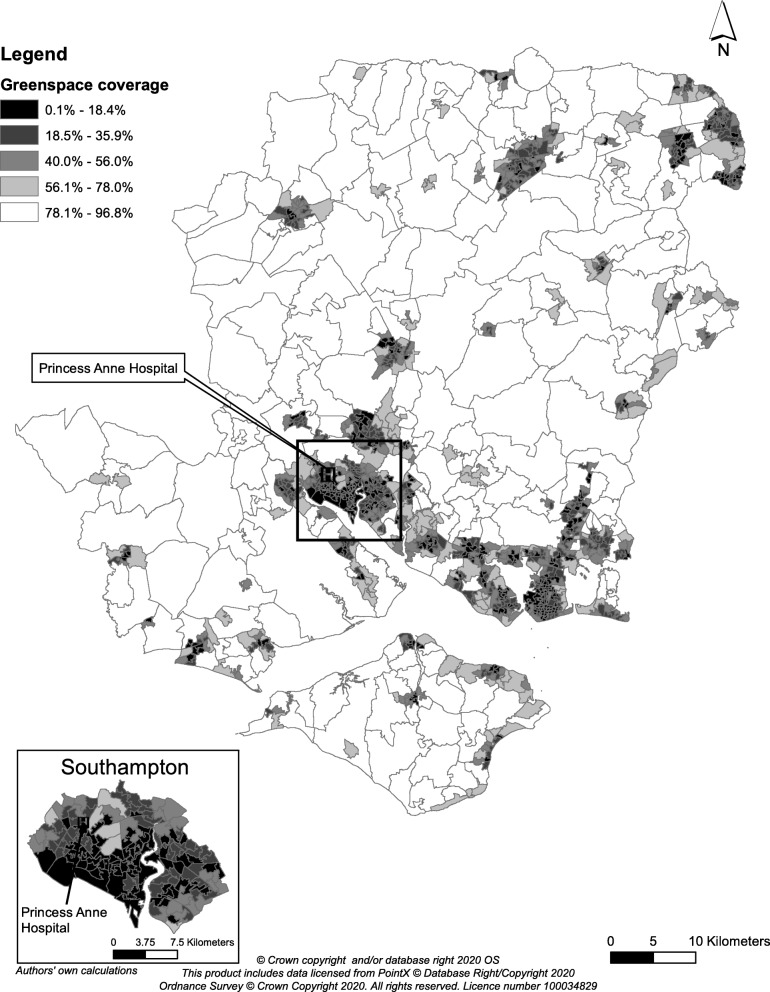
Greenspace coverage across LSOAs across Southampton and the surrounding county of Hampshire, 2006

The map for greenspace coverage in 2006 for the city of Southampton and the surrounding county at the LSOA level is displayed in Fig. 3. All maps for the distribution of area characteristics and their change over time for LSOAs are in Additional file 1. The southern area of Southampton (where the city centre is located) had lower greenspace coverage, but higher walkability, supermarket density, relative density of unhealthy food outlets and particulate matter levels than the outskirts of the city, and in comparison with the remainder of the county of Hampshire. NO_*x*_ levels were somewhat even within the city, with lower levels in the east of the city. Over time, the city and surrounding county became more obesogenic, with greenspace coverage, supermarket density, particulate matter and NO_*x*_ levels decreasing, whilst the relative density of unhealthy food outlets tended to increase, particularly in the south of the city.

Table 2 displays the risk ratios (RRs) for each area characteristic and the risk of being affected by overweight or obesity at ages 4–5 and 10–11. There is limited evidence of any association between area characteristics at birth and weight at ages 4–5, and all factors attenuate after adjustment for individual-level confounding.

**Table 2:** Risk ratios for area characteristics at birth and their associations with overweight or obesity at ages 4–5 and 10–11 in Southampton, UK

Area factor	Scale	Model	Age 4–5				Age 10–11			
			All [*n* = 14,084]		Movers [*n* = 2772]		All [*n* = 5637]		Movers [*n* = 2965]	
			RR	95% CI	RR	95% CI	RR	95% CI	RR	95% CI
Greenspace (%)	LSOA*	Unadjusted	1.000	0.998–1.002	1.001	0.998–1.004	**0.997**	**0.996–0.999**	0.997	0.994–1.000
		Adjusted	0.999	0.997–1.001	1.000	0.997–1.003	**0.997**	**0.995–0.999**	**0.994**	**0.990–0.999**
	MSOA*	Unadjusted	0.999	0.997–1.001	1.001	0.997–1.005	**0.996**	**0.994–0.998**	**0.995**	**0.991–0.999**
		Adjusted	1.000	0.998–1.002	1.000	0.997–1.004	**0.997**	**0.995–0.999**	0.995	0.989–1.000
Walkability	LSOA*	Unadjusted	1.008	0.995–1.021	1.014	0.991–1.038	**1.016**	**1.001–1.031**	1.013	0.991–1.035
		Adjusted	1.006	0.994–1.017	1.019	0.998–1.042	1.009	0.995–1.022	1.019	0.984–1.056
	MSOA*	Unadjusted	1.008	0.998–1.019	1.006	0.982–1.031	**1.022**	**1.007–1.037**	1.019	0.995–1.045
		Adjusted	1.004	0.992–1.015	1.011	0.987–1.036	1.012	0.996–1.028	1.015	0.981–1.050
Supermarket density [a]	LSOA*	Unadjusted	1.010	0.964–1.057	1.007	0.926–1.095	**0.938**	**0.886–0.993**	0.989	0.871–1.122
		Adjusted	1.015	0.976–1.056	1.010	0.933–1.094	0.959	0.914–1.005	1.011	0.915–1.117
	MSOA*	Unadjusted	1.022	0.957–1.091	1.063	0.950–1.188	0.960	0.894–1.030	0.981	0.804–1.198
		Adjusted	1.018	0.972–1.066	1.056	0.950–1.173	0.972	0.916–1.032	1.038	0.900–1.197
Relative density of unhealthy food outlets [b]	LSOA*	Unadjusted	1.002	0.994–1.009	1.001	0.989–1.012	**1.019**	**1.006–1.031**	**1.029**	**1.003–1.056**
		Adjusted	1.000	0.993–1.007	1.002	0.992–1.013	1.009	0.998–1.020	1.020	0.994–1.046
	MSOA*	Unadjusted	0.998	0.984–1.011	0.998	0.981–1.016	**1.033**	**1.016–1.051**	**1.046**	**1.005–1.088**
		Adjusted	0.997	0.987–1.008	1.001	0.985–1.016	**1.021**	**1.005–1.037**	1.019	0.982–1.057
Spaces for social interaction	LSOA*	Unadjusted	1.004	0.998–1.011	1.005	0.994–1.016	**1.009**	**1.001–1.016**	1.006	0.995–1.017
		Adjusted	1.002	0.996–1.008	1.006	0.997–1.016	1.000	0.993–1.008	1.007	0.989–1.026
	MSOA*	Unadjusted	**1.009**	**1.001–1.018**	1.007	0.990–1.024	**1.015**	**1.007–1.024**	**1.017**	**1.002–1.032**
		Adjusted	1.006	0.999–1.013	1.011	0.997–1.025	1.004	0.993–1.016	1.008	0.984–1.034
PM_2.5_	LSOA*	Unadjusted	0.982	0.960–1.005	0.972	0.931–1.015	1.008	0.977–1.039	0.999	0.956–1.044
		Adjusted	0.988	0.967–1.010	0.984	0.944–1.027	1.007	0.978–1.038	1.044	0.973–1.120
	MSOA*	Unadjusted	**0.980**	**0.960–1.000**	0.963	0.925–1.002	1.008	0.976–1.041	0.996	0.952–1.042
		Adjusted	0.984	0.964–1.005	0.978	0.939–1.018	1.008	0.980–1.037	1.042	0.965–1.125
PM_10_	LSOA*	Unadjusted	0.989	0.975–1.004	0.977	0.951–1.003	1.021	0.997–1.045	1.023	0.990–1.056
		Adjusted	0.994	0.980–1.008	0.984	0.959–1.009	1.017	0.994–1.041	1.028	0.978–1.080
	MSOA*	Unadjusted	0.988	0.974–1.002	**0.971**	**0.947–0.995**	1.021	1.000–1.044	1.021	0.989–1.055
		Adjusted	0.992	0.979–1.005	0.978	0.955–1.002	**1.019**	**1.001–1.036**	1.028	0.975–1.085
NO_*x*_	LSOA*	Unadjusted	1.000	0.997–1.003	0.998	0.993–1.003	1.004	0.997–1.010	1.006	0.998–1.014
		Adjusted	1.000	0.997–1.002	0.998	0.993–1.003	1.001	0.995–1.007	0.994	0.977–1.010
	MSOA*	Unadjusted	1.000	0.997–1.002	0.997	0.992–1.001	1.003	0.995–1.012	1.003	0.990–1.016
		Adjusted	0.999	0.997–1.001	0.997	0.993–1.002	1.002	0.995–1.009	0.987	0.970–1.005

*LSOA* areas with average populations of 1500 and an area of 4 km^2^, *MSOA* areas with average populations of 7000 and an area of 21 km^2^. All models adjust for clustering of observations within areas. Adjusted models control for maternal BMI and smoking in early pregnancy, educational attainment, ethnicity and parity, but not other area characteristics. [a] All results adjusted for the relative density of unhealthy food outlets. [b] All results adjusted for supermarket density. Relative risk ratios with *p* < 0.05 are in bold

In unadjusted models, greenspace and supermarket density (LSOA only) were negatively associated with the risk of being overweight or obese at ages 10–11, whereas walkability, the food index and spaces for social interaction were positively associated. After adjusting for confounding, a 1% increase in the LSOA covered by greenspace remained negatively associated with being overweight or obese (RR 0.997, 95% confidence interval [CI] 0.995–0.999), with a similar effect size at the MSOA level. The relative density of unhealthy food outlets (adjusted for supermarket density) remained positively associated with childhood weight (RR 1.021, 95% CI 1.005–1.037) at the MSOA level, but not the LSOA level. A 1-μg/m^3^ increase in the annual average exposure to PM_10_ became associated with being overweight or obese after adjustment for confounding (RR 1.019, 95% CI 1.001–1.036) at the MSOA level, but not the LSOA level. Of these findings, only greenspace at the LSOA level was significantly associated with the sub-sample of children who changed home LSOA before age 10–11 (RR 0.994, 95% CI 0.990–0.999). We were unable to additionally adjust for siblings being clustered within mothers, because our approach does not support multiple levels of clustering. To account for this, a sensitivity analysis was undertaken where the sample was restricted to one child per mother, and the results were broadly similar (Additional file 2). The effect sizes for the environmental characteristics at the LSOA level were also broadly similar in complete case and multiple imputation analyses for the year 6 outcome (for greenspace RR 0.998, 95% CI 0.997–1.000). (Additional file 3). We have based our interpretation and conclusion on the complete case analyses.

## Discussion

Findings from this analysis suggest that access to greenspace in early life is associated with a lower risk of becoming overweight or obese by age 10–11. This association was scale dependent, where it was observed for all children and movers at smaller administrative areas (LSOA, average area 4 km^2^) but not for movers at the larger administrative scale (MSOA, average area 21 km^2^).

The relative density of unhealthy food outlets and PM_10_ exposure were associated with an increased risk of overweight or obese by age 10–11, but this finding was not replicated when restricted to movers. Other area characteristics either presented limited evidence of an association or the associations did not persist in the sample of children who moved. Differences in the associations between environmental area characteristics and childhood weight for children who moved and those who stayed have been highlighted in previous research [45]. This suggests that the duration of exposure may be important, rather than the level of exposure specifically, and data with greater temporal detail are required to clarify this issue.

Several studies have theorised that greenspace is related to childhood weight because these environments facilitate or make physical activity more attractive [5]. This may explain why we find that greenspace is associated with childhood weight at ages 10–11 but not 4–5, because children gain more autonomy over their activity spaces as they become older [46], and thus, greenspace may become more facilitative of physical activity for older children. There may be other explanations for this association, however. Functional greenspaces such as parks and playing fields are positively associated with house prices in Great Britain [47], and house prices may act as a proxy for family income, which in turn affects the healthiness of the child’s diet [48]. In the same analysis, total greenspace (including non-functional greenspace such as shrubbery) was negatively associated with house prices [47], so the direction may depend on how broad the definition of greenspace is. Natural greenspaces encourage physical activity and offer opportunities for social interaction for women in pregnancy which may offset stress levels [49], which in turn affect placental endocrine and immune processes that affect offspring susceptibility to overweight and obesity [50]. Greenspace access has been shown to be associated with other area factors before, wherein greenspace offsets air pollution [51]. It could be a combination of these correlated factors that explain the protective effect, rather than greenspace in isolation.

In comparison with previous longitudinal research in this area [14], there were some differences in the findings. A one standard deviation increase in NO_*x*_ exposure in the first year of life was associated with an increase in BMI of 0.5 units at age 10–11 previously [52], whereas there was no evidence of an association in this study. This could be explained by the difference in units of exposure, wherein we utilised a 1-μg/m^3^ change in exposure, and the standard deviation of NO_*x*_ amounts to 12.6 μg/m^3^ in our sample. A previous systematic review highlighted two studies that found a negative association between early-life PM_10_ exposure and weight in childhood, one study found a positive association and one study demonstrated no association, where we observed an association among children at age 10–11 at the MSOA level [14]. Walkability in childhood was not shown to be associated with childhood weight before [53]. The relative density of unhealthy food outlets has been associated cross-sectionally with higher BMI among children in the UK [54], and we are the first to reaffirm that finding longitudinally over time for overweight and obesity in this study, but this association was not found among children who moved. In previous studies, as we have shown here, the effect sizes of area-level influences are small relative to the effect of individual-level characteristics in explaining child weight status. There is a possibility that positive or negative area-level characteristics are clustered, as seen in the recent area-based neighbourhood classification of neighbourhoods, Access to Healthy Assets and Hazards or the Indices of Deprivation [55]. The aim of our research was to understand what aspects of a local environment may influence child weight status later in life—just as ‘good’ or ‘detrimental’ individual characteristics may be concentrated for some individuals, the same may be true of neighbourhoods.

The concept of ‘health-promoting environments’ has attracted policy attention as a means to reduce the prevalence of non-communicable diseases such as obesity. For example, goal 11.7 of the World Health Organization’s sustainable development goals is to provide universal access to green and public spaces [56]. The findings of this study provide further evidence that access to greenspace can be a potential policy-level intervention for the early prevention of overweight and obesity among children. The UK Government’s National Planning Policy Framework already stipulates that housing applications should provide accessibility of open spaces [57], and this could be used to protect greenspace access for future generations. That is not to state that factors such as the food environment and air pollution are not important for child health, but rather, we did not observe a longitudinal association for these factors at birth and subsequent childhood weight above and beyond confounding factors such as migration, maternal BMI and education in our study population.

The findings of this study should be interpreted in the context of its strengths and limitations. This study benefits from a large dataset of routine data, which reduces the chance that the sample is not representative of the local population. We have attempted to account for population migration affecting the study results through a sub-analysis of children who moved home LSOA between birth and weight measurement. The child’s weight and height were measured by trained personnel [22], increasing the validity of reliability of these measures in comparison with self-reported data. With the exception of walkability, all area characteristics were measured on an annual basis, increasing the relevance of the estimated exposure at birth for participants in this study. Under- or over-adjustment for individual factors can lead to bias in estimates in the effects of area characteristics on childhood weight [58]. This analysis addresses these issues through constructing a DAG [59] which explicitly details assumptions about the mechanisms through which commonly used areal factors influence childhood weight, and how these are related to maternal and child individual-level confounders.

This study has limitations related to the data used. There was a large proportion of children with missing outcome data. As the addresses of children were aggregated at the LSOA level, there will be misclassification in exposure levels than if we had exact address data. The population in this study is limited to children born within a regional hospital who then lived in the catchment area for one NHS Trust which covers the city of Southampton, UK, and those living in the suburban regions outside the city. Southampton is a provincial urban city which is more deprived than the average local authority in England, as is common with cities [60], but the city has a similar social class distribution to England [61]. We did succeed in linking hospital antenatal data to children attending schools in the county of Hampshire (in which Southampton is situated), but historical LSOA data were not available to the research team. There were several key confounders that we could not adjust for, including paternal factors, maternal diet in pregnancy, parental diet and physical activity, family income, child’s diet and physical activity. All of these factors are likely related to the types of areas children reside in [4], which could impact the estimation of associations between area characteristics and the development of overweight or obesity. In relation to the food environment assessment, there is potential for misclassification in delineating ‘healthy’ and ‘unhealthy’ food outlets, as some varieties of stores provide health foods alongside calorie-dense foods, for example, bakeries.

This is an observational study, and it is not possible to ascribe causal effects to area characteristics. Intervention studies are not feasible on this scale, and thus, natural experiments (comparisons between children within areas that experience change) are the ‘gold standard’ in this area [34]. Natural experiments of area change are biased by population migration, however, because several neighbourhood regeneration programmes have led to prior residents becoming priced out of these areas, resulting in such schemes not improving the environments of low-income residents [62].

## Conclusions

This study suggests that the amount of greenspace in the local area at birth is negatively associated with the risk of becoming affected by overweight or obesity at the end of primary school, even in those children who potentially have a change in exposure to greenspace after birth. Increasing access to greenspace during preconception, pregnancy and early years can be a policy-level intervention aimed at the early prevention of childhood obesity.

## Supplementary information

**Additional file 1.** Maps for area characteristics and their change over time for 2011 boundaries for Lower layer Super Output Areas.

**Additional file 2.** Sensitivity analysis comparing an analysis on all children, and an analysis restricted to one child per mother.

**Additional file 3.** Sensitivity analysis comparing the results between complete case analysis and multiple imputation for the outcome at 10-11 years.

## Data Availability

The data owners are University Hospital Southampton NHS Trust, Solent NHS Trust, and Ordnance Survey. Anonymised data are only available upon request from the PI (NAA) conditional on the approval of the appropriate institutional ethics, research governance processes and data holders.

## Abbreviations

BMI: Body mass index
DAG: Directed acyclic graph
LSOA: Lower layer Super Output Areas
MSOA: Middle layer Super Output Areas
NCMP: National Childhood Measurement Programme
NHS: National Health Service
PM: Particulate matter
RR: Risk ratio
UHS: University Hospital Southampton
