# Supplementary material for: Are environmental area characteristics at birth associated with overweight and obesity in school-aged children? Findings from the SLOPE (Studying Lifecourse Obesity PrEdictors) population-based cohort in the south of England
Source: BMC Med. 2020 Mar 19;18:43. doi: 10.1186/s12916-020-01513-0 (PMC7081603; doi:10.1186/s12916-020-01513-0)
Supplement: Supplementary file 1 — Additional file 1. Maps for area characteristics and their change over time for 2011 boundaries for Lower layer Super Output Areas. [file 12916_2020_1513_MOESM1_ESM.pdf]

Title: Are environmental area characteristics at birth associated with overweight and obesity in school-aged children? Findings from the SLOPE (Studying Lifecourse Obesity PrEdictors) population-based cohort in the south of England

Additional file 1 – Maps for area characteristics and their change over time for 2011 boundaries for Lower layer Super Output Areas

Authors' own calculations

© Crown copyright and/or database right 2020 OS
This product includes data licensed from PointX © Database Right/Copyright 2020
Ordnance Survey © Crown Copyright 2020. All rights reserved. Licence number 100034829

Greenspace coverage across Hampshire (2006), LSOAs

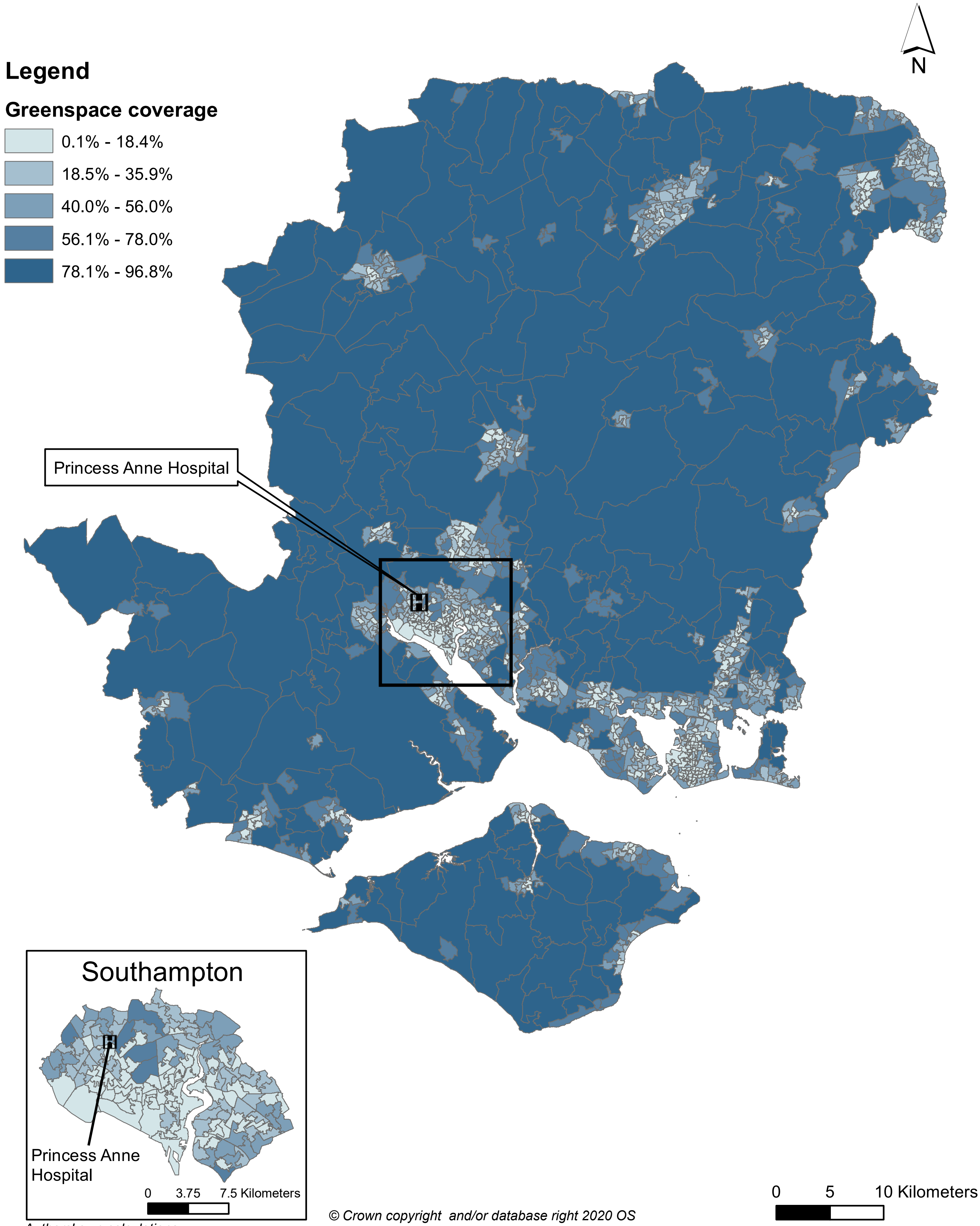

Authors' own calculations

© Crown copyright and/or database right 2020 OS
This product includes data licensed from PointX © Database Right/Copyright 2020
Ordnance Survey © Crown Copyright 2020. All rights reserved. Licence number 100034829

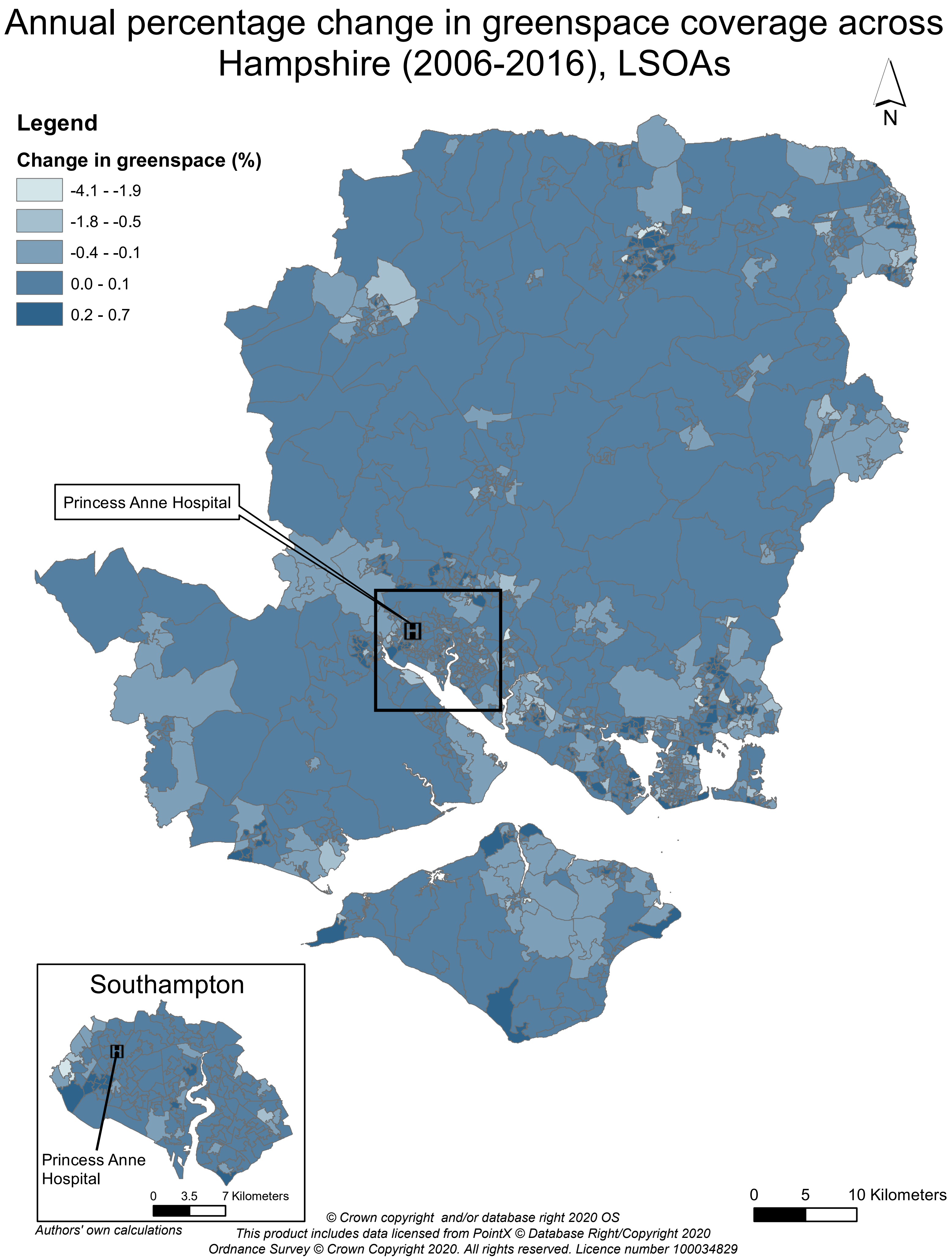

Annual percentage change in greenspace coverage across Hampshire (2006-2016), LSOAs
Legend
Change in greenspace (%)
-4.1 - -1.9
-1.8 - -0.5
-0.4 - -0.1
0.0 - 0.1
0.2 - 0.7
N
Princess Anne Hospital
Southampton
Princess Anne Hospital
0 3.5 7 Kilometers
0 5 10 Kilometers
Authors' own calculations
© Crown copyright and/or database right 2020 OS
This product includes data licensed from PointX © Database Right/Copyright 2020
Ordnance Survey © Crown Copyright 2020. All rights reserved. Licence number 100034829

Walkability index in Hampshire, LSOAs

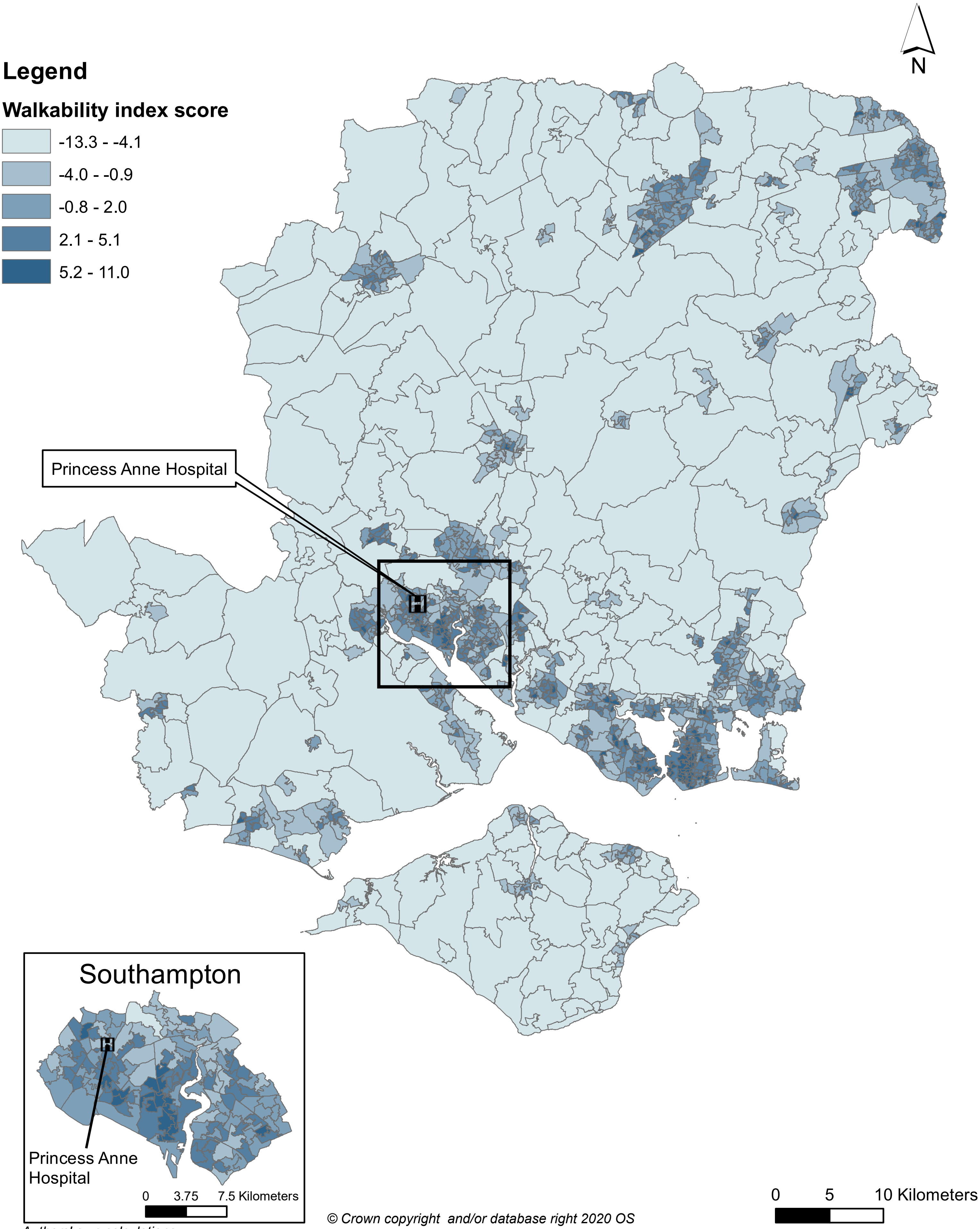

Authors' own calculations

© Crown copyright and/or database right 2020 OS
This product includes data licensed from PointX © Database Right/Copyright 2020
Ordnance Survey © Crown Copyright 2020. All rights reserved. Licence number 100034829

Average supermarket density within 800m across Hampshire (2007), LSOAs

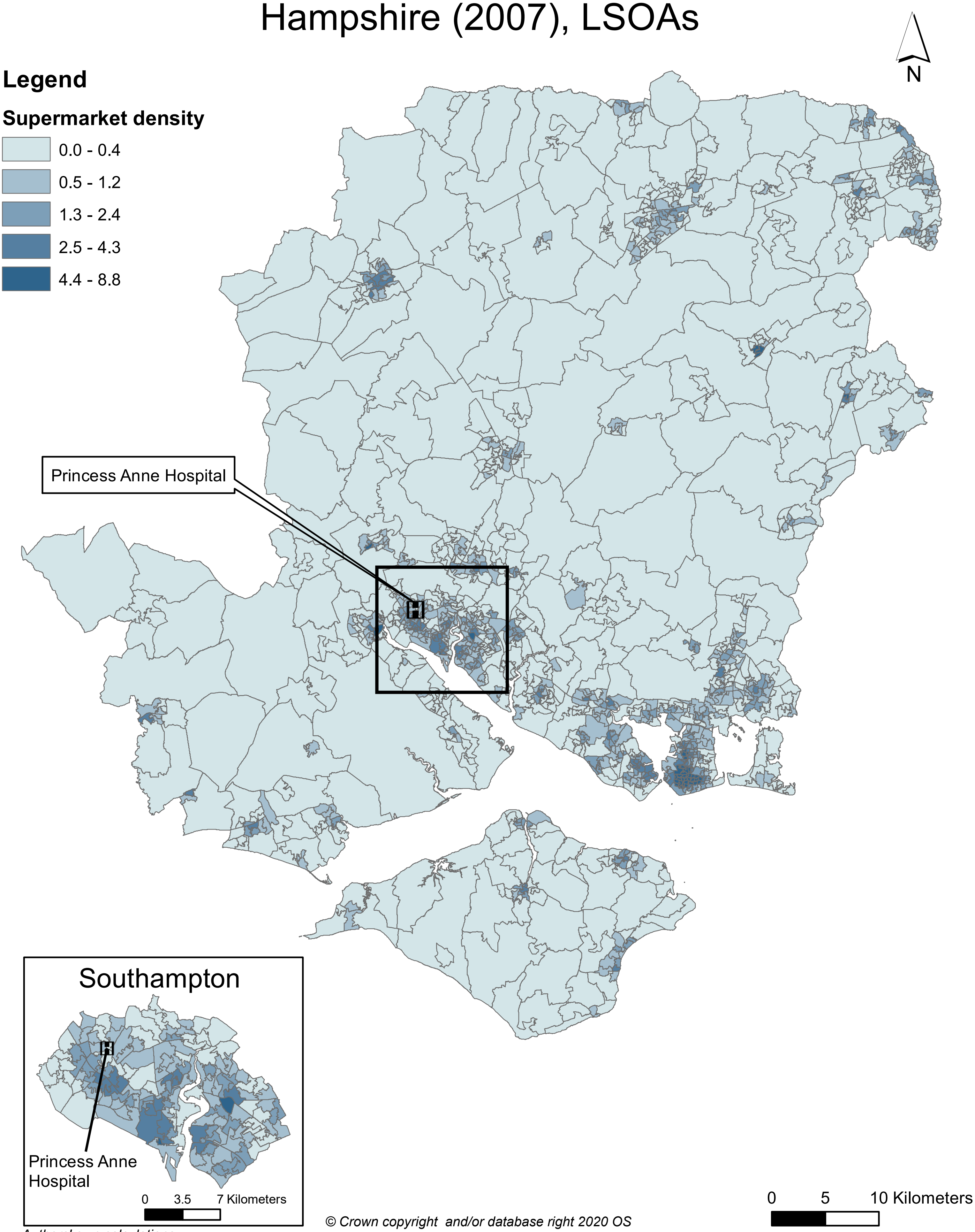

Authors' own calculations

© Crown copyright and/or database right 2020 OS
This product includes data licensed from PointX © Database Right/Copyright 2020
Ordnance Survey © Crown Copyright 2020. All rights reserved. Licence number 100034829

Annual percentage change in supermarket density across Hampshire (2007-2017), LSOAs

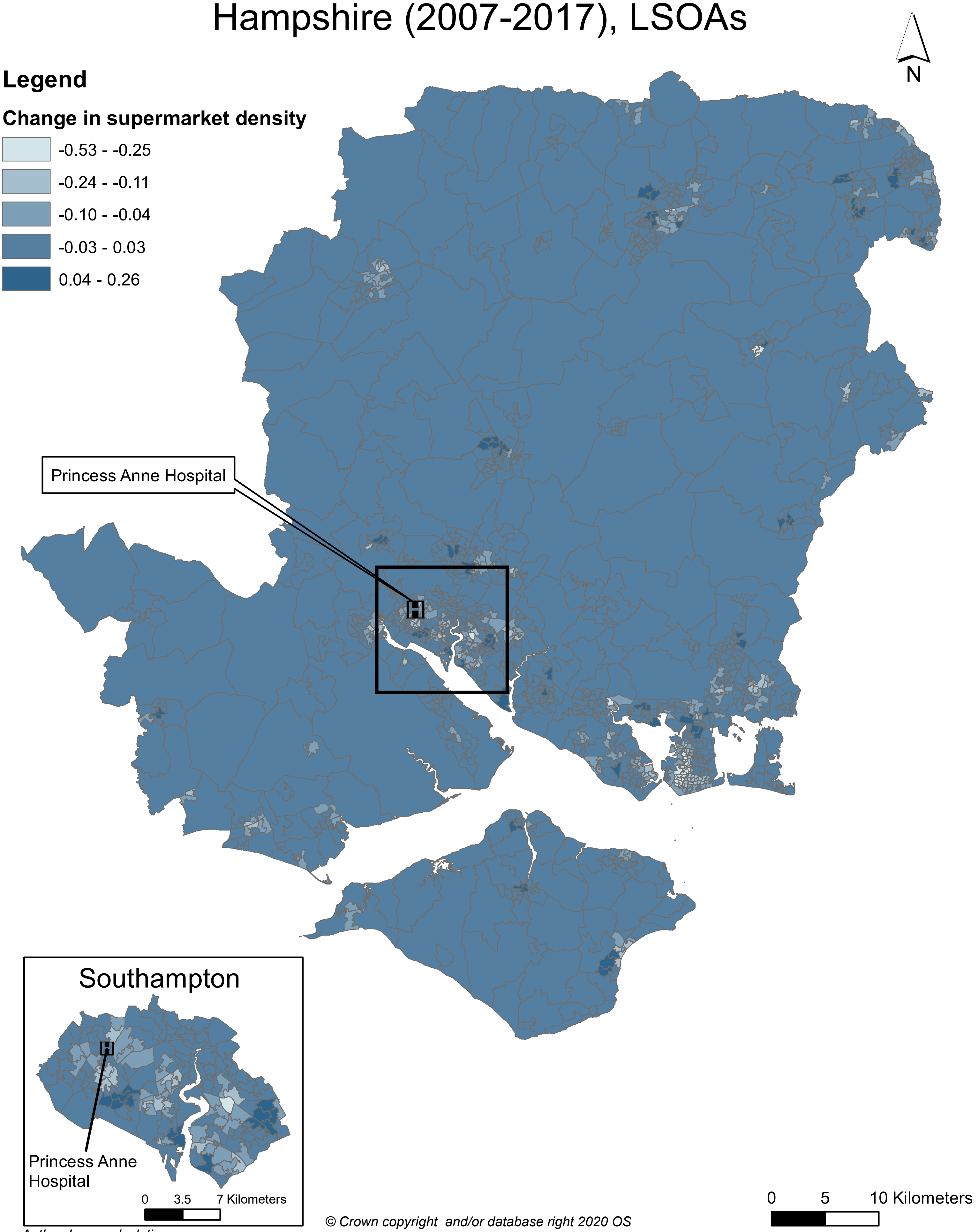

Authors' own calculations

© Crown copyright and/or database right 2020 OS
This product includes data licensed from PointX © Database Right/Copyright 2020
Ordnance Survey © Crown Copyright 2020. All rights reserved. Licence number 100034829

Unhealthy food index in Hampshire (2007), LSOAs

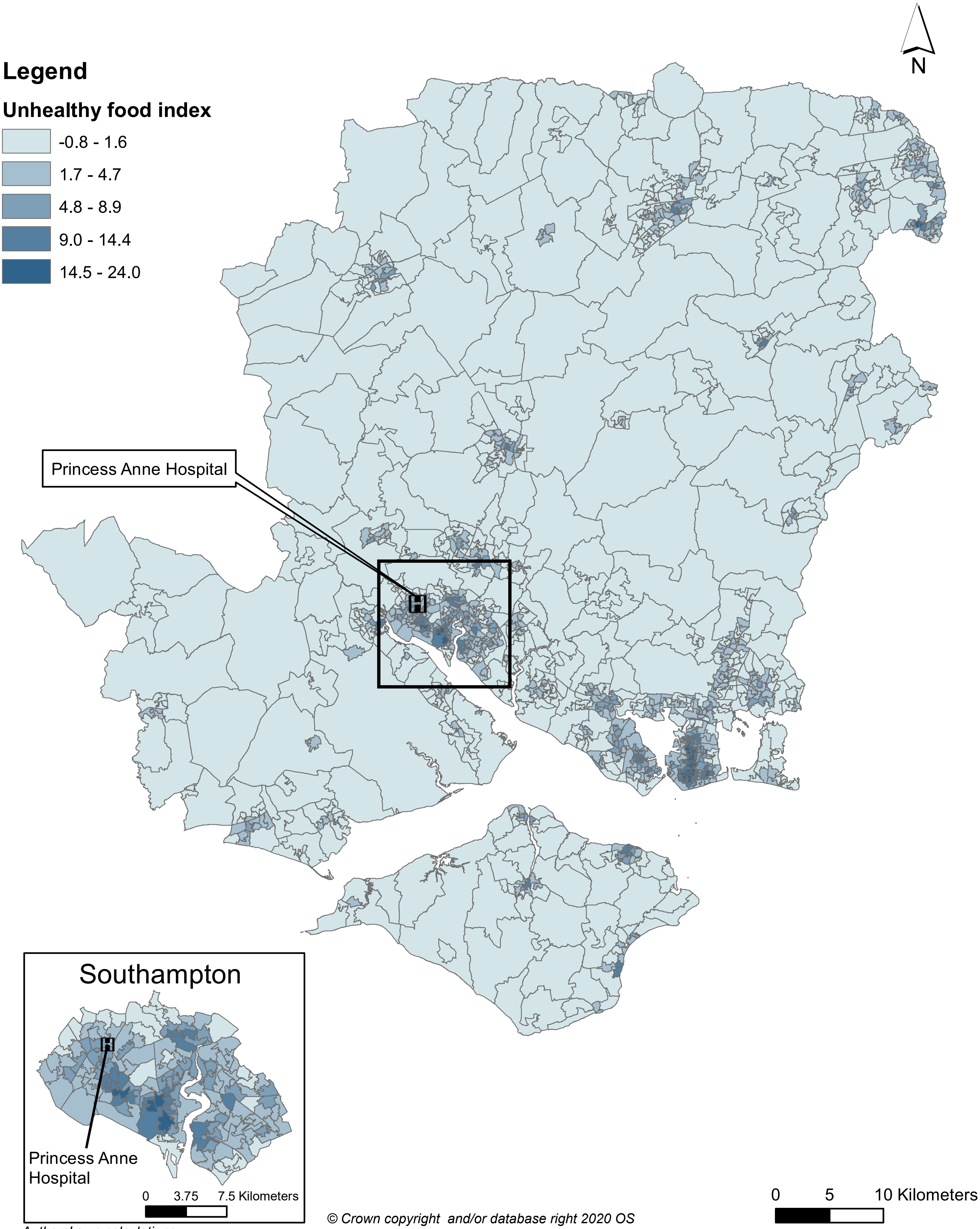

Authors' own calculations

© Crown copyright and/or database right 2020 OS
This product includes data licensed from PointX © Database Right/Copyright 2020
Ordnance Survey © Crown Copyright 2020. All rights reserved. Licence number 100034829

Change in unhealthy food index in Hampshire (2007-2017), LSOAs

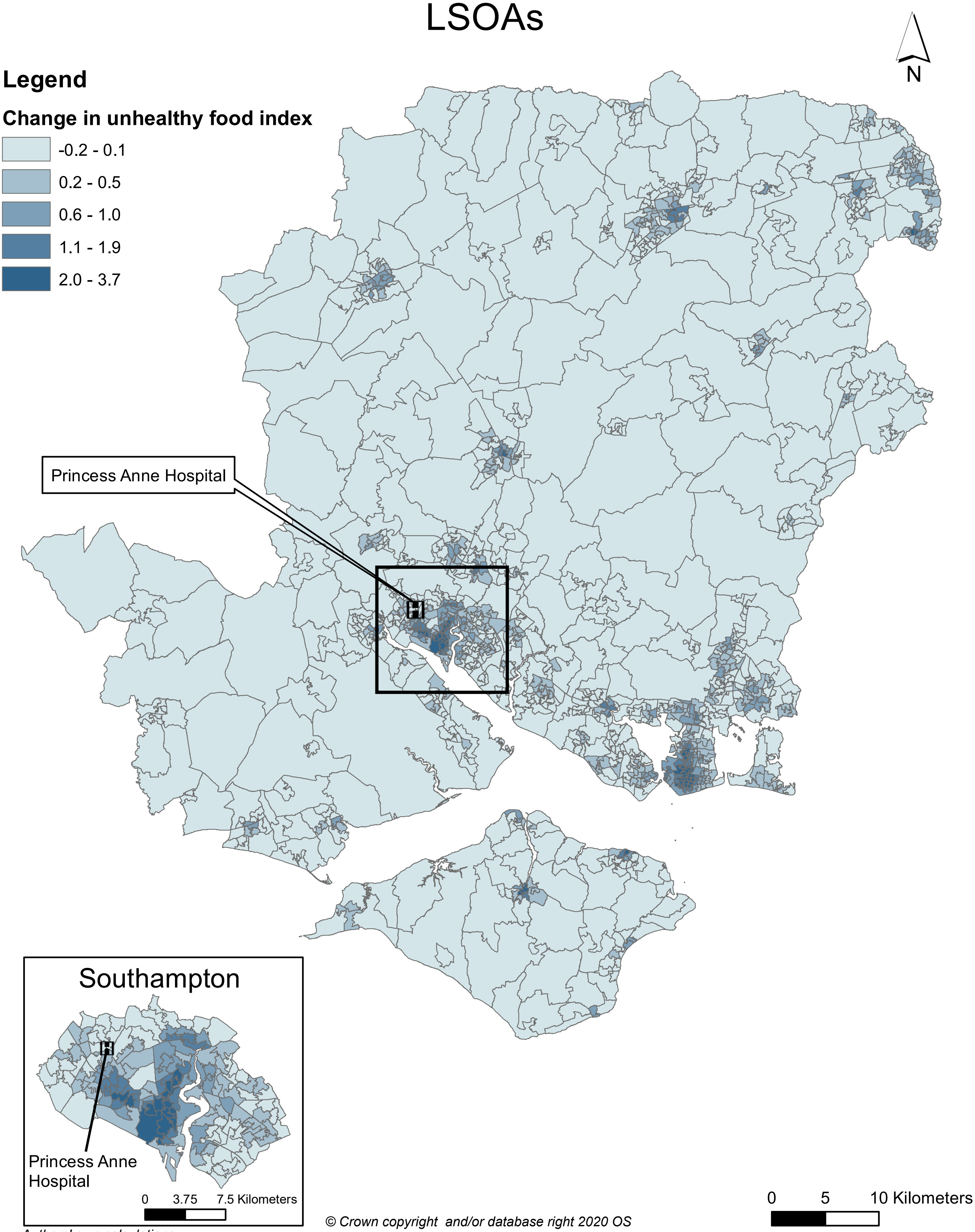

Authors' own calculations

© Crown copyright and/or database right 2020 OS
This product includes data licensed from PointX © Database Right/Copyright 2020
Ordnance Survey © Crown Copyright 2020. All rights reserved. Licence number 100034829

Average spaces for social interaction density within 800m across Hampshire (2007), LSOAs

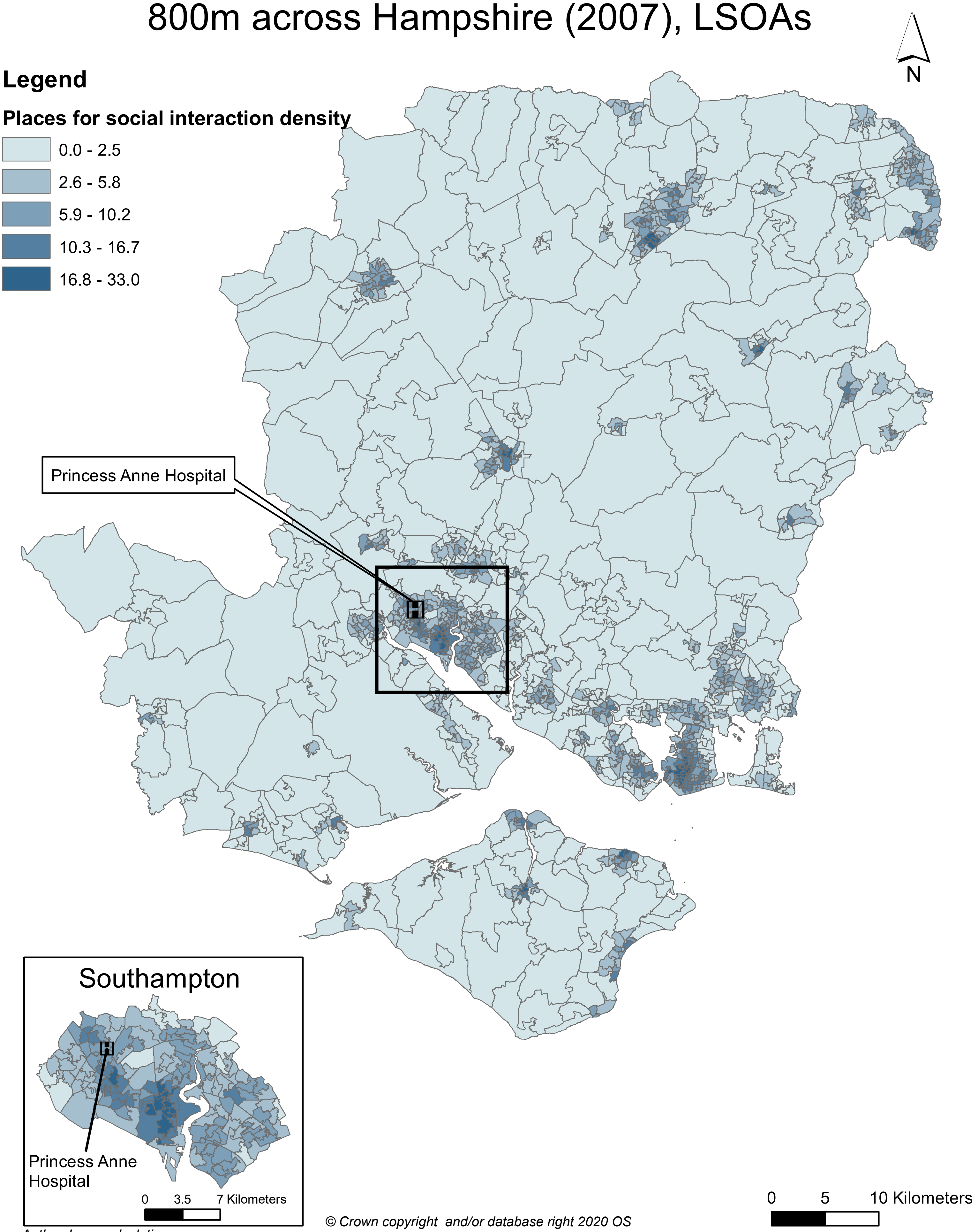

Authors' own calculations

© Crown copyright and/or database right 2020 OS
This product includes data licensed from PointX © Database Right/Copyright 2020
Ordnance Survey © Crown Copyright 2020. All rights reserved. Licence number 100034829

Annual change in average spaces for social interaction density within 800m across Hampshire (2007-2017), LSOAs

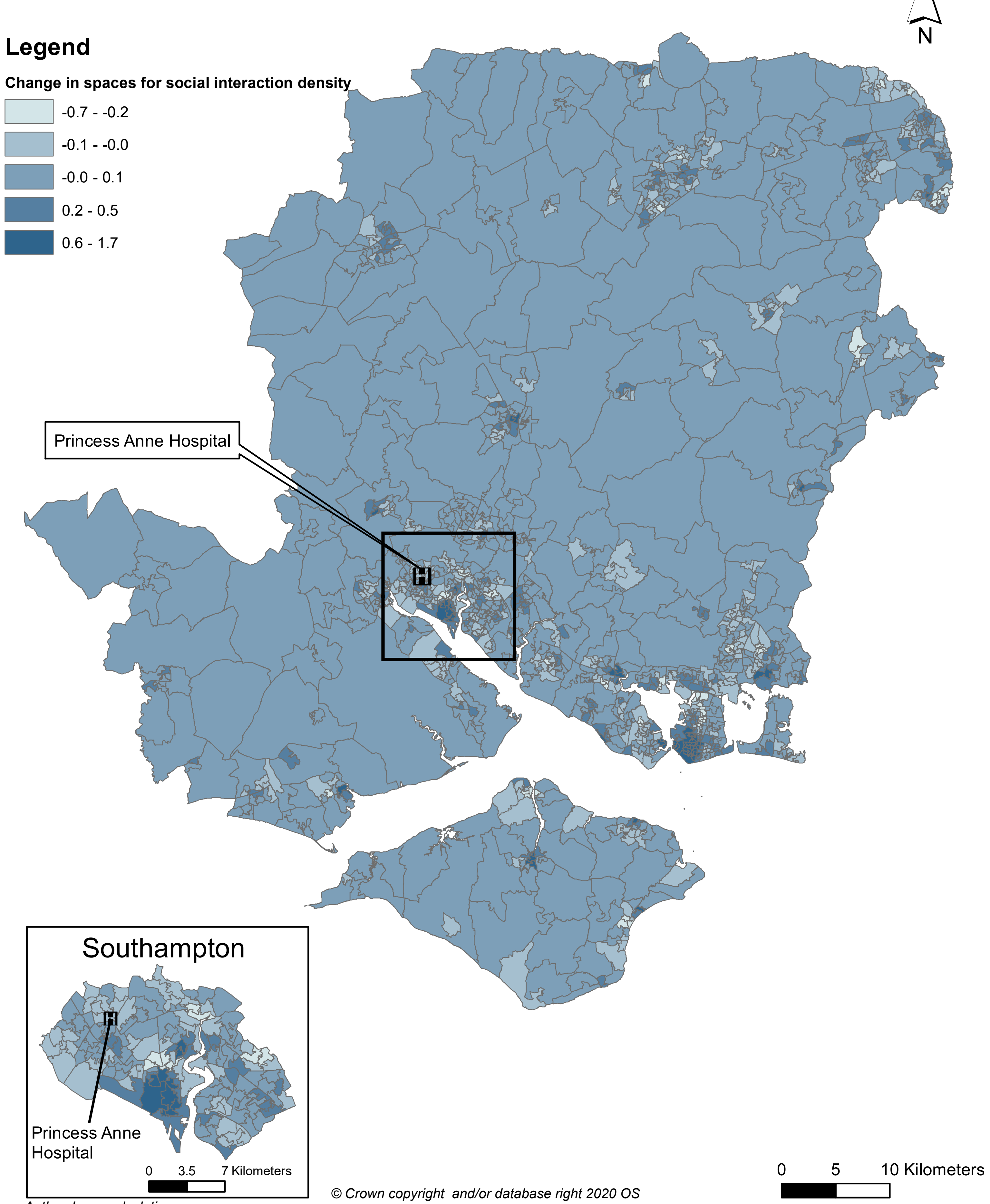

Authors' own calculations

© Crown copyright and/or database right 2020 OS
This product includes data licensed from PointX © Database Right/Copyright 2020
Ordnance Survey © Crown Copyright 2020. All rights reserved. Licence number 100034829

Annual average particulate matter (<2.5 μm) in μg/m^3 across Hampshire (2003), LSOAs

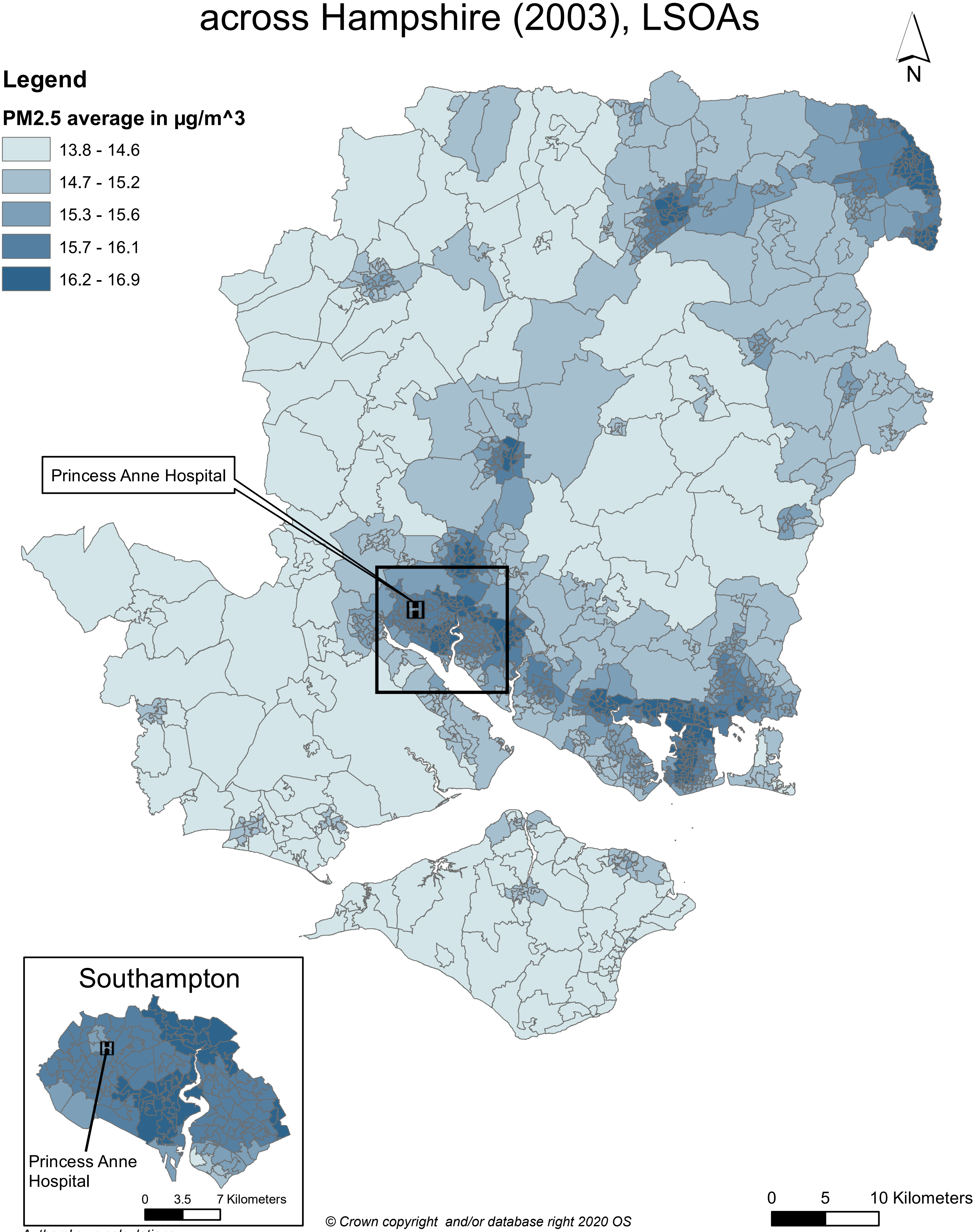

Authors' own calculations

© Crown copyright and/or database right 2020 OS

This product includes data licensed from PointX © Database Right/Copyright 2020

Ordnance Survey © Crown Copyright 2020. All rights reserved. Licence number 100034829

Average change in annual average particulate matter (<2.5 μm) in μg/m^3 across Hampshire (2003-2017), LSOAs

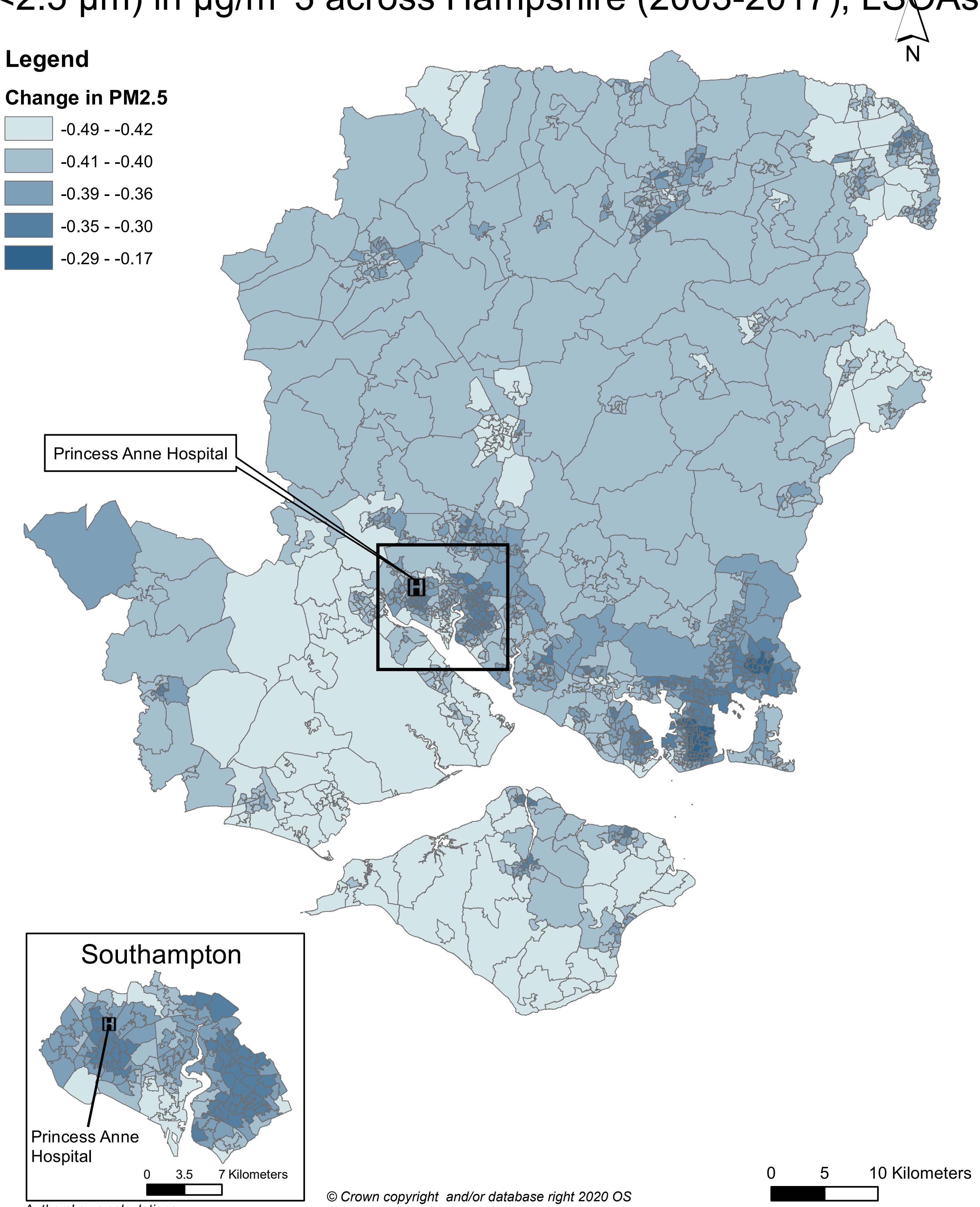

Authors' own calculations

© Crown copyright and/or database right 2020 OS
This product includes data licensed from PointX © Database Right/Copyright 2020
Ordnance Survey © Crown Copyright 2020. All rights reserved. Licence number 100034829

Annual average particulate matter (<10 μm) in μg/m^3 across Hampshire (2003), LSOAs

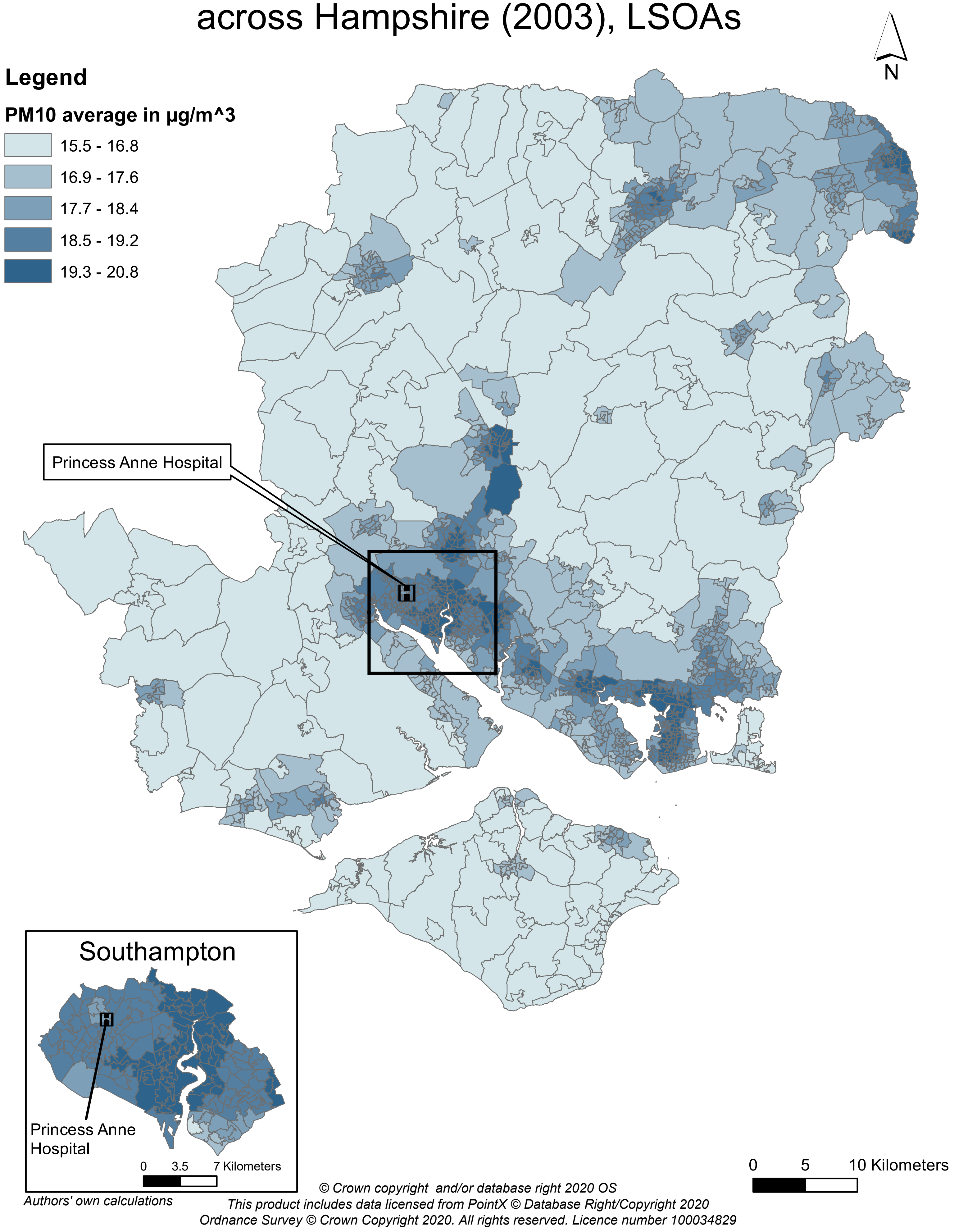

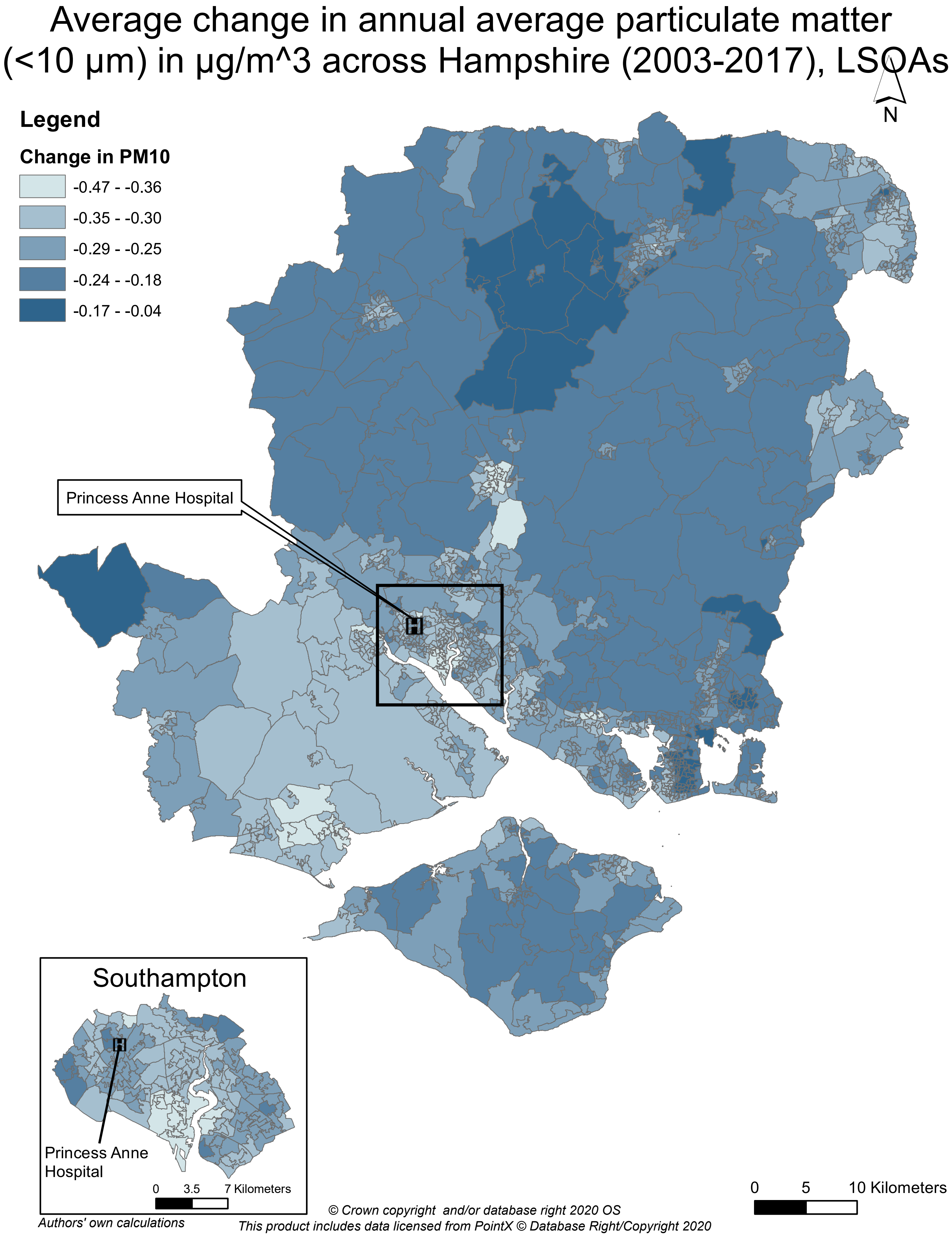

© Crown copyright and/or database right 2020 OS
This product includes data licensed from PointX © Database Right/Copyright 2020
Ordnance Survey © Crown Copyright 2020. All rights reserved. Licence number 100034829

Annual average nitrogen oxides in µg/m^3 across Hampshire (2003), LSOAs

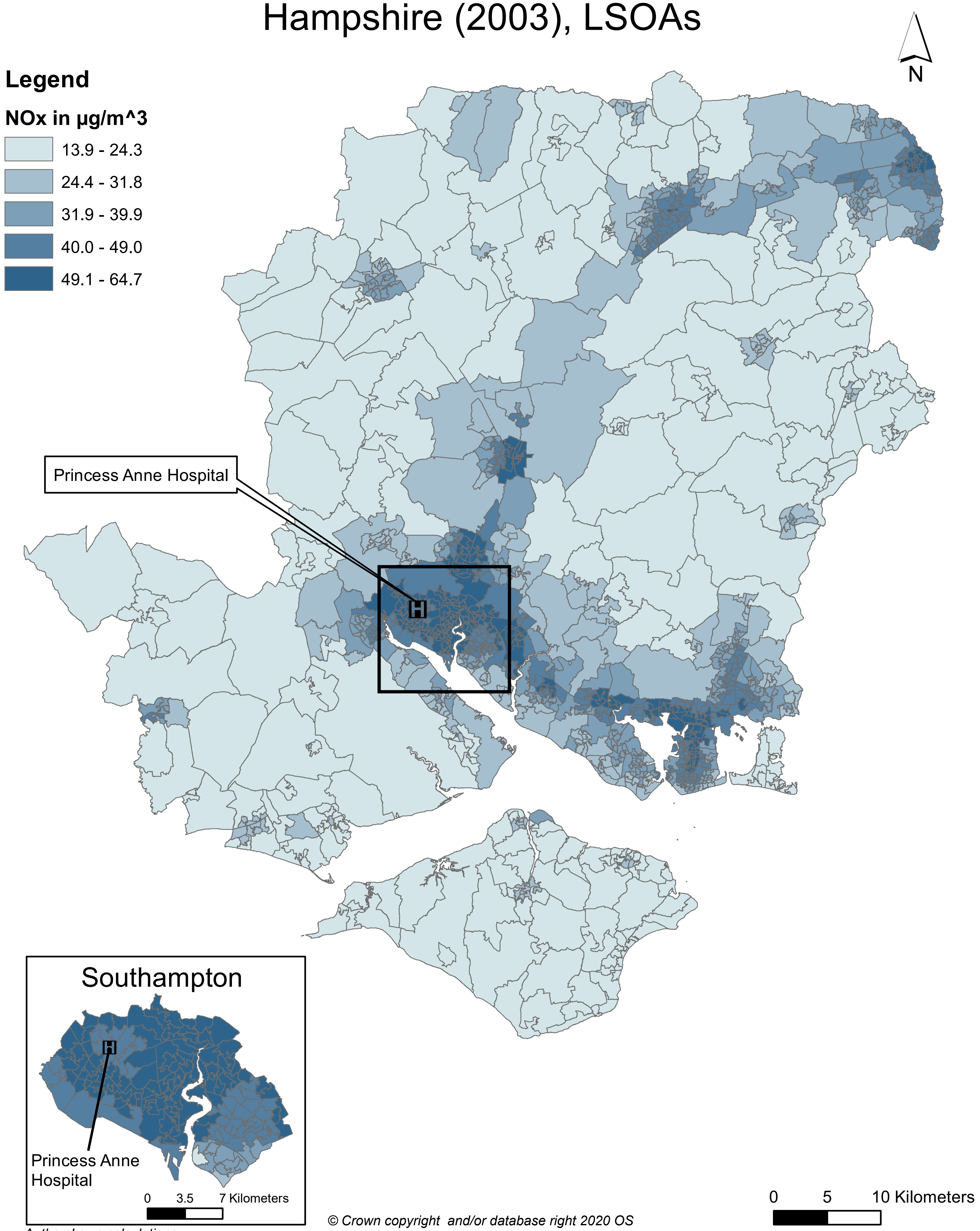

Authors' own calculations

© Crown copyright and/or database right 2020 OS
This product includes data licensed from PointX © Database Right/Copyright 2020
Ordnance Survey © Crown Copyright 2020. All rights reserved. Licence number 100034829

Average change in annual average nitrogen oxides in µg/m^3 across Hampshire (2003-2017), LSOAs

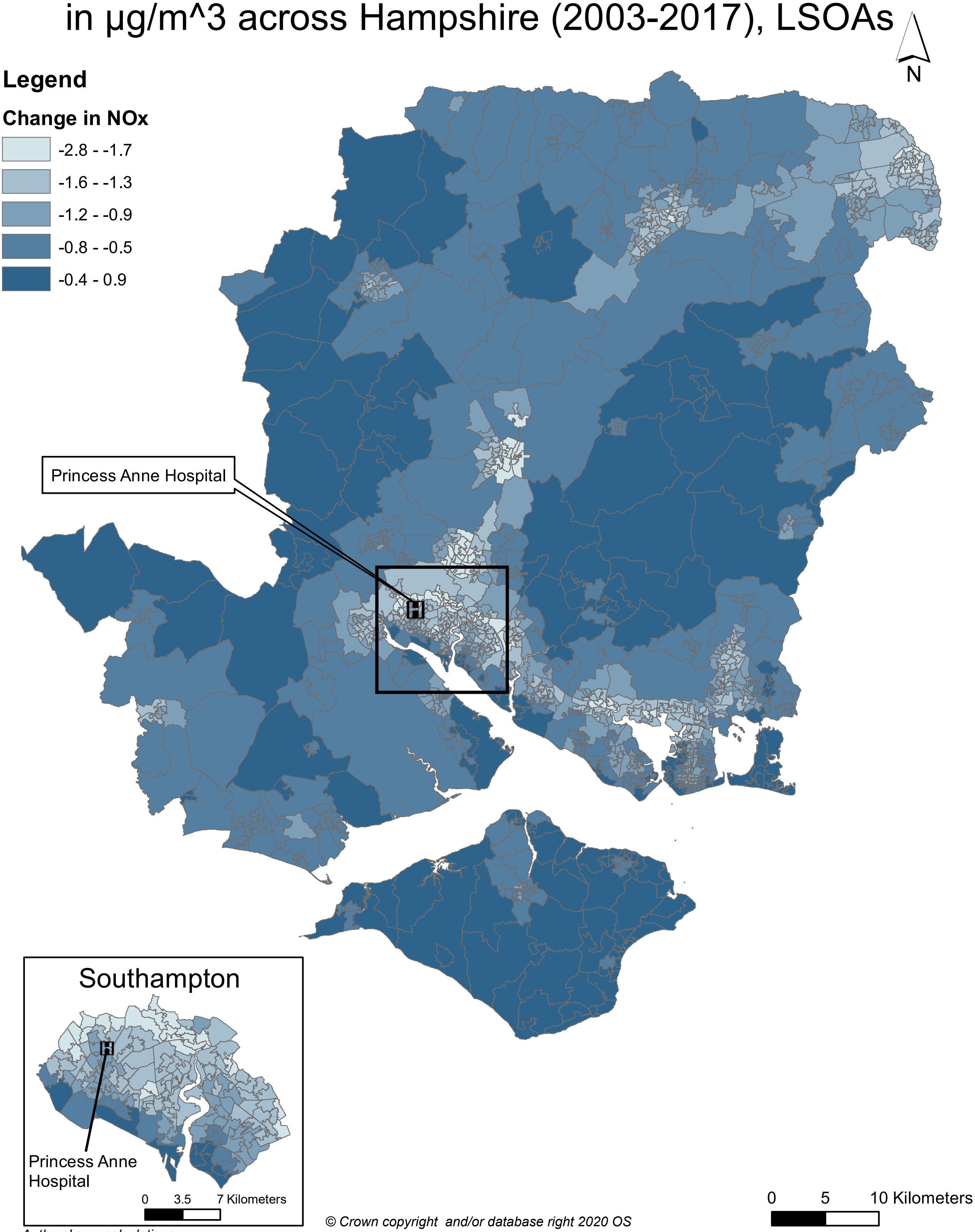

Authors' own calculations

© Crown copyright and/or database right 2020 OS
This product includes data licensed from PointX © Database Right/Copyright 2020
Ordnance Survey © Crown Copyright 2020. All rights reserved. Licence number 100034829
