# Supplementary material for: Are environmental area characteristics at birth associated with overweight and obesity in school-aged children? Findings from the SLOPE (Studying Lifecourse Obesity PrEdictors) population-based cohort in the south of England
Source: BMC Med. 2020 Mar 19;18:43. doi: 10.1186/s12916-020-01513-0 (PMC7081603; doi:10.1186/s12916-020-01513-0)
Supplement: Supplementary file 2 — Additional file 2. Sensitivity analysis comparing an analysis on all children, and an analysis restricted to one child per mother. [file 12916_2020_1513_MOESM2_ESM.docx]

**Title: Are environmental area characteristics at birth associated with overweight and obesity in school-aged children? Findings from the SLOPE (Studying Lifecourse Obesity PrEdictors) population-based cohort in the south of England**

**Additional file 2 – sensitivity analysis comparing an analysis on all children, and an analysis restricted to one child per mother**

**Table S1 – Relative risk ratios for area characteristics at birth and their associations with overweight or obesity at age 4-5 in the SLOPE cohort which were linked to address data, including a sub-sample restricted to one child per mother**

|  |  |  |  | | | | | | | |
| --- | --- | --- | --- | --- | --- | --- | --- | --- | --- | --- |
|  |  |  | All | | | | Movers | | | |
|  |  |  | Multiple observations per mother | | One observation per mother | | Multiple observations per mother | | One observation per mother | |
| Area factor | Scale | Model | RRR | 95% CI | RRR | 95% CI | RRR | 95% CI | RRR | 95% CI |
| Greenspace (%) | LSOA* | Unadjusted | 1.000 | 0.998 - 1.002 | 1.000 | 0.998 - 1.002 | 1.001 | 0.998 - 1.004 | 1.002 | 0.998 - 1.006 |
|  |  | Adjusted | 0.999 | 0.997 - 1.001 | 0.999 | 0.997 - 1.001 | 1.000 | 0.997 - 1.003 | 1.001 | 0.997 - 1.004 |
|  | MSOA* | Unadjusted | 0.999 | 0.997 - 1.001 | 0.999 | 0.997 - 1.001 | 1.001 | 0.997 - 1.005 | 1.001 | 0.996 - 1.005 |
|  |  | Adjusted | 1.000 | 0.998 - 1.002 | 0.999 | 0.997 - 1.001 | 1.000 | 0.997 - 1.004 | 1.000 | 0.996 - 1.004 |
| Walkability | LSOA* | Unadjusted | 1.008 | 0.995 - 1.021 | 1.011 | 0.997 - 1.026 | 1.014 | 0.991 - 1.038 | 1.011 | 0.984 - 1.039 |
|  |  | Adjusted | 1.006 | 0.994 - 1.017 | 1.011 | 0.998 - 1.023 | 1.019 | 0.998 - 1.042 | 1.018 | 0.992 - 1.045 |
|  | MSOA* | Unadjusted | 1.008 | 0.998 - 1.019 | 1.010 | 0.999 - 1.021 | 1.006 | 0.982 - 1.031 | 1.007 | 0.983 - 1.032 |
|  |  | Adjusted | 1.004 | 0.992 - 1.015 | 1.006 | 0.995 - 1.017 | 1.011 | 0.987 - 1.036 | 1.013 | 0.990 - 1.037 |
| Supermarket density [a] | LSOA* | Unadjusted | 1.010 | 0.964 - 1.057 | 1.014 | 0.966 - 1.066 | 1.007 | 0.926 - 1.095 | 1.001 | 0.907 - 1.106 |
|  |  | Adjusted | 1.015 | 0.976 - 1.056 | 1.019 | 0.973 - 1.066 | 1.010 | 0.933 - 1.094 | 0.998 | 0.904 - 1.101 |
|  | MSOA* | Unadjusted | 1.022 | 0.957 - 1.091 | 1.044 | 0.984 - 1.108 | 1.063 | 0.950 - 1.188 | 1.071 | 0.956 - 1.201 |
|  |  | Adjusted | 1.018 | 0.972 - 1.066 | 1.038 | 0.997 - 1.080 | 1.056 | 0.950 - 1.173 | 1.060 | 0.957 - 1.175 |
| Food index [b] | LSOA* | Unadjusted | 1.002 | 0.994 - 1.009 | 1.001 | 0.994 - 1.009 | 1.001 | 0.989 - 1.012 | 1.001 | 0.988 - 1.013 |
|  |  | Adjusted | 1.000 | 0.993 - 1.007 | 1.000 | 0.993 - 1.007 | 1.002 | 0.992 - 1.013 | 1.003 | 0.992 - 1.014 |
|  | MSOA* | Unadjusted | 0.998 | 0.984 - 1.011 | 0.997 | 0.985 - 1.009 | 0.998 | 0.981 - 1.016 | 0.999 | 0.981 - 1.018 |
|  |  | Adjusted | 0.997 | 0.987 - 1.008 | 0.996 | 0.987 - 1.005 | 1.001 | 0.985 - 1.016 | 1.003 | 0.988 - 1.017 |
| Spaces for social interaction | LSOA* | Unadjusted | 1.004 | 0.998 - 1.011 | 1.003 | 0.995 - 1.011 | 1.005 | 0.994 - 1.016 | 1.003 | 0.990 - 1.015 |
|  |  | Adjusted | 1.002 | 0.996 - 1.008 | 1.000 | 0.993 - 1.007 | 1.006 | 0.997 - 1.016 | 1.004 | 0.993 - 1.015 |
|  | MSOA* | Unadjusted | **1.009** | **1.001 - 1.018** | **1.009** | **1.000 - 1.018** | 1.007 | 0.990 - 1.024 | 1.007 | 0.989 - 1.025 |
|  |  | Adjusted | 1.006 | 0.999 - 1.013 | 1.005 | 0.997 - 1.013 | 1.011 | 0.997 - 1.025 | 1.010 | 0.996 - 1.025 |
| PM2.5 | LSOA* | Unadjusted | 0.982 | 0.960 - 1.005 | 0.987 | 0.963 - 1.012 | 0.972 | 0.931 - 1.015 | 0.976 | 0.932 - 1.023 |
|  |  | Adjusted | 0.988 | 0.967 - 1.010 | 0.992 | 0.969 - 1.017 | 0.984 | 0.944 - 1.027 | 0.989 | 0.946 - 1.035 |
|  | MSOA* | Unadjusted | **0.980** | **0.960 - 1.000** | 0.984 | 0.966 - 1.003 | 0.963 | 0.925 - 1.002 | 0.969 | 0.924 - 1.016 |
|  |  | Adjusted | 0.984 | 0.964 - 1.005 | 0.988 | 0.967 - 1.009 | 0.978 | 0.939 - 1.018 | 0.984 | 0.939 - 1.031 |
| PM10 | LSOA* | Unadjusted | 0.989 | 0.975 - 1.004 | 0.994 | 0.979 - 1.009 | 0.977 | 0.951 - 1.003 | 0.984 | 0.956 - 1.013 |
|  |  | Adjusted | 0.994 | 0.980 - 1.008 | 0.998 | 0.983 - 1.012 | 0.984 | 0.959 - 1.009 | 0.992 | 0.967 - 1.019 |
|  | MSOA* | Unadjusted | 0.988 | 0.974 - 1.002 | 0.992 | 0.979 - 1.005 | **0.971** | **0.947 - 0.995** | 0.979 | 0.953 - 1.006 |
|  |  | Adjusted | 0.992 | 0.979 - 1.005 | 0.995 | 0.983 - 1.008 | 0.978 | 0.955 - 1.002 | 0.987 | 0.961 - 1.015 |
| NOx | LSOA* | Unadjusted | 1.000 | 0.997 - 1.003 | 1.000 | 0.997 - 1.003 | 0.998 | 0.993 - 1.003 | 0.996 | 0.989 - 1.002 |
|  |  | Adjusted | 1.000 | 0.997 - 1.002 | 1.000 | 0.997 - 1.003 | 0.998 | 0.993 - 1.003 | 0.996 | 0.990 - 1.002 |
|  | MSOA* | Unadjusted | 1.000 | 0.997 - 1.002 | 0.999 | 0.997 - 1.002 | 0.997 | 0.992 - 1.001 | 0.995 | 0.990 - 1.000 |
|  |  | Adjusted | 0.999 | 0.997 - 1.001 | 0.999 | 0.997 - 1.001 | 0.997 | 0.993 - 1.002 | 0.995 | 0.991 - 1.000 |
| LSOA = areas with average populations of 1,500; MSOA = areas with average populations of 7,000. All models adjust for clustering of observations within areas. Adjusted models control for maternal BMI and smoking in early pregnancy, education, ethnicity and parity. [a] all results adjusted for the unhealthy food index. [b] all results adjusted for supermarket density. | | | | | | | | | | |

**Table S2 – Relative risk ratios for area characteristics at birth and their associations with overweight or obesity at age 10-11 in the SLOPE cohort which were linked to address data, including a sub-sample restricted to one child per mother**

|  |  |  |  | | | | | | | |
| --- | --- | --- | --- | --- | --- | --- | --- | --- | --- | --- |
|  |  |  | All | | | | Movers | | | |
|  |  |  | Multiple observations per mother | | One observation per mother | | Multiple observations per mother | | One observation per mother | |
| Area factor | Scale | Model | RRR | 95% CI | RRR | 95% CI | RRR | 95% CI | RRR | 95% CI |
| Greenspace (%) | LSOA* | Unadjusted | **0.997** | **0.996 - 0.999** | **0.998** | **0.996 - 1.000** | 0.997 | 0.994 - 1.000 | 0.997 | 0.994 - 1.000 |
|  |  | Adjusted | **0.997** | **0.995 - 0.999** | **0.997** | **0.995 - 0.999** | **0.994** | **0.990 - 0.999** | **0.995** | **0.990 - 0.999** |
|  | MSOA* | Unadjusted | **0.996** | **0.994 - 0.998** | **0.996** | **0.994 - 0.998** | **0.995** | **0.991 - 0.999** | **0.995** | **0.991 - 0.999** |
|  |  | Adjusted | **0.997** | **0.995 - 0.999** | **0.997** | **0.995 - 0.999** | 0.995 | 0.989 - 1.000 | **0.994** | **0.989 - 0.999** |
| Walkability | LSOA* | Unadjusted | 1.016 | 1.001 - 1.031 | 1.015 | 1.000 - 1.031 | 1.013 | 0.991 - 1.035 | 1.013 | 0.990 - 1.036 |
|  |  | Adjusted | 1.009 | 0.995 - 1.022 | 1.010 | 0.997 - 1.024 | 1.019 | 0.984 - 1.056 | 1.016 | 0.980 - 1.053 |
|  | MSOA* | Unadjusted | **1.022** | **1.007 - 1.037** | **1.023** | **1.007 - 1.039** | 1.019 | 0.995 - 1.045 | 1.020 | 0.994 - 1.046 |
|  |  | Adjusted | 1.012 | 0.996 - 1.028 | 1.015 | 0.998 - 1.031 | 1.015 | 0.981 - 1.050 | 1.021 | 0.989 - 1.054 |
| Supermarket density [a] | LSOA* | Unadjusted | 0.938 | 0.886 - 0.993 | 0.942 | 0.885 - 1.002 | 0.989 | 0.871 - 1.122 | 0.999 | 0.868 - 1.149 |
|  |  | Adjusted | 0.959 | 0.914 - 1.005 | 0.963 | 0.917 - 1.011 | 1.011 | 0.915 - 1.117 | 1.015 | 0.910 - 1.132 |
|  | MSOA* | Unadjusted | 0.960 | 0.894 - 1.030 | 0.955 | 0.887 - 1.028 | 0.981 | 0.804 - 1.198 | 0.975 | 0.793 - 1.198 |
|  |  | Adjusted | 0.972 | 0.916 - 1.032 | 0.969 | 0.911 - 1.030 | 1.038 | 0.900 - 1.197 | 1.036 | 0.912 - 1.178 |
| Food index [b] | LSOA* | Unadjusted | **1.019** | **1.006 - 1.031** | **1.018** | **1.004 - 1.032** | **1.029** | **1.003 - 1.056** | 1.024 | 0.996 - 1.054 |
|  |  | Adjusted | 1.009 | 0.998 - 1.020 | 1.008 | 0.996 - 1.020 | 1.020 | 0.994 - 1.046 | 1.016 | 0.989 - 1.044 |
|  | MSOA* | Unadjusted | **1.033** | **1.016 - 1.051** | **1.036** | **1.017 - 1.055** | **1.046** | **1.005 - 1.088** | **1.049** | **1.005 - 1.095** |
|  |  | Adjusted | **1.021** | **1.005 - 1.037** | **1.024** | **1.008 - 1.040** | 1.019 | 0.982 - 1.057 | 1.023 | 0.993 - 1.054 |
| Spaces for social interaction | LSOA* | Unadjusted | **1.009** | **1.001 - 1.016** | **1.009** | **1.001 - 1.017** | 1.006 | 0.995 - 1.017 | 1.005 | 0.995 - 1.016 |
|  |  | Adjusted | 1.000 | 0.993 - 1.008 | 1.002 | 0.995 - 1.010 | 1.007 | 0.989 - 1.026 | 1.011 | 0.992 - 1.030 |
|  | MSOA* | Unadjusted | **1.015** | **1.007 - 1.024** | **1.016** | **1.007 - 1.025** | **1.017** | **1.002 - 1.032** | **1.016** | **1.001 - 1.031** |
|  |  | Adjusted | 1.004 | 0.993 - 1.016 | 1.006 | 0.995 - 1.018 | 1.008 | 0.984 - 1.034 | 1.014 | 0.993 - 1.036 |
| PM2.5 | LSOA* | Unadjusted | 1.008 | 0.977 - 1.039 | 1.007 | 0.974 - 1.040 | 0.999 | 0.956 - 1.044 | 1.002 | 0.956 - 1.051 |
|  |  | Adjusted | 1.007 | 0.978 - 1.038 | 1.004 | 0.973 - 1.036 | 1.044 | 0.973 - 1.120 | 1.036 | 0.966 - 1.112 |
|  | MSOA* | Unadjusted | 1.008 | 0.976 - 1.041 | 1.007 | 0.976 - 1.040 | 0.996 | 0.952 - 1.042 | 0.998 | 0.952 - 1.047 |
|  |  | Adjusted | 1.008 | 0.980 - 1.037 | 1.004 | 0.976 - 1.033 | 1.042 | 0.965 - 1.125 | 1.031 | 0.954 - 1.114 |
| PM10 | LSOA* | Unadjusted | 1.021 | 0.997 - 1.045 | 1.021 | 0.996 - 1.046 | 1.023 | 0.990 - 1.056 | 1.023 | 0.987 - 1.060 |
|  |  | Adjusted | 1.017 | 0.994 - 1.041 | 1.017 | 0.993 - 1.041 | 1.028 | 0.978 - 1.080 | 1.021 | 0.971 - 1.074 |
|  | MSOA* | Unadjusted | 1.021 | 1.000 - 1.044 | 1.021 | 0.999 - 1.044 | 1.021 | 0.989 - 1.055 | 1.021 | 0.985 - 1.058 |
|  |  | Adjusted | 1.019 | 1.001 - 1.036 | 1.017 | 0.999 - 1.036 | 1.028 | 0.975 - 1.085 | 1.019 | 0.964 - 1.078 |
| NOx | LSOA* | Unadjusted | 1.004 | 0.997 - 1.010 | 1.004 | 0.997 - 1.011 | 1.006 | 0.998 - 1.014 | 1.007 | 0.998 - 1.016 |
|  |  | Adjusted | 1.001 | 0.995 - 1.007 | 1.002 | 0.995 - 1.009 | 0.994 | 0.977 - 1.010 | 0.994 | 0.979 - 1.009 |
|  | MSOA* | Unadjusted | 1.003 | 0.995 - 1.012 | 1.003 | 0.995 - 1.012 | 1.003 | 0.990 - 1.016 | 1.004 | 0.991 - 1.016 |
|  |  | Adjusted | 1.002 | 0.995 - 1.009 | 1.002 | 0.995 - 1.009 | 0.987 | 0.970 - 1.005 | 0.988 | 0.971 - 1.005 |
| LSOA = areas with average populations of 1,500; MSOA = areas with average populations of 7,000. All models adjust for clustering of observations within areas. Adjusted models control for maternal BMI and smoking in early pregnancy, educational attainment, ethnicity and parity. [a] all results adjusted for the unhealthy food index. [b] all results adjusted for supermarket density. | | | | | | | | | | |
