# Supplementary material for: Are environmental area characteristics at birth associated with overweight and obesity in school-aged children? Findings from the SLOPE (Studying Lifecourse Obesity PrEdictors) population-based cohort in the south of England
Source: BMC Med. 2020 Mar 19;18:43. doi: 10.1186/s12916-020-01513-0 (PMC7081603; doi:10.1186/s12916-020-01513-0)
Supplement: Supplementary file 3 — Additional file 3. Sensitivity analysis comparing the results between complete case analysis and multiple imputation for the outcome at 10-11 years. [file 12916_2020_1513_MOESM3_ESM.docx]

**Title: Are environmental area characteristics at birth associated with overweight and obesity in school-aged children? Findings from the SLOPE (Studying Lifecourse Obesity PrEdictors) population-based cohort in the south of England**

**Additional file 3 – sensitivity analysis comparing the results between complete case analysis and multiple imputation for the outcome at 10-11 years**

**Table S3 – Risk ratios for area characteristics at birth and their associations with overweight or obesity at age 10-11, Southampton, UK: Comparison of complete case analysis [n = 5,637] and multiple imputation for the outcome [n = 11,208]**

|  |  |  | Complete case analysis [n = 5,637] | | | | Multiple Imputation [n = 11,208] | | | |
| --- | --- | --- | --- | --- | --- | --- | --- | --- | --- | --- |
| Area factor | Scale | Model | RR | p | 95% CI LB | 95% CI UB | RR | p | 95% CI LB | 95% CI UB |
| Greenspace (%) | LSOA | Unadjusted | **0.997** | **0.012** | **0.996** | **0.999** | **0.998** | **0.046** | **0.997** | **1.000** |
|  |  | Adjusted | **0.997** | **0.002** | **0.995** | **0.999** | 0.998 | 0.062 | 0.997 | 1.000 |
| Walkability | LSOA | Unadjusted | **1.016** | **0.033** | **1.001** | **1.031** | 1.011 | 0.067 | 0.999 | 1.023 |
|  |  | Adjusted | 1.009 | 0.207 | 0.995 | 1.022 | 1.005 | 0.367 | 0.994 | 1.016 |
| Supermarket density [a] | LSOA | Unadjusted | **0.938** | **0.029** | **0.886** | **0.993** | 0.966 | 0.130 | 0.923 | 1.010 |
|  |  | Adjusted | 0.959 | 0.081 | 0.914 | 1.005 | 0.981 | 0.369 | 0.942 | 1.023 |
| Relative density of unhealthy food outlets [b] | LSOA | Unadjusted | **1.019** | **0.003** | **1.006** | **1.031** | **1.012** | **0.027** | **1.001** | **1.023** |
|  |  | Adjusted | 1.009 | 0.120 | 0.998 | 1.020 | 1.005 | 0.370 | 0.995 | 1.015 |
| Spaces for social interaction | LSOA | Unadjusted | **1.009** | **0.022** | **1.001** | **1.016** | **1.007** | **0.036** | **1.000** | **1.013** |
|  |  | Adjusted | 1.000 | 0.917 | 0.993 | 1.008 | 1.001 | 0.780 | 0.994 | 1.008 |
| PM_2.5_ | LSOA | Unadjusted | 1.008 | 0.636 | 0.977 | 1.039 | 1.003 | 0.817 | 0.981 | 1.024 |
|  |  | Adjusted | 1.007 | 0.640 | 0.978 | 1.038 | 1.001 | 0.899 | 0.981 | 1.022 |
| PM_10_ | LSOA | Unadjusted | 1.021 | 0.089 | 0.997 | 1.045 | 1.008 | 0.371 | 0.991 | 1.026 |
|  |  | Adjusted | 1.017 | 0.150 | 0.994 | 1.041 | 1.008 | 0.360 | 0.991 | 1.025 |
| NO_x_ | LSOA | Unadjusted | 1.004 | 0.261 | 0.997 | 1.010 | 1.002 | 0.421 | 0.998 | 1.006 |
|  |  | Adjusted | 1.001 | 0.767 | 0.995 | 1.007 | 1.000 | 0.922 | 0.996 | 1.004 |
| LSOA (Lower Super Output Area)= areas with average populations of 1,500 and an area of 4 km^2^. All models adjust for clustering of observations within areas. Adjusted models control for maternal BMI and smoking in early pregnancy, education, ethnicity and parity, but not other area characteristics. [a] all results adjusted for the relative density of unhealthy food outlets. [b] all results adjusted for supermarket density. Relative risk ratios with p<0.05 are in **bold**. | | | | | | | | | | |
